# Unraveling the complexity of rat object vision requires a full convolutional network and beyond

**DOI:** 10.1016/j.patter.2024.101149

**Published:** 2025-01-17

**Authors:** Paolo Muratore, Alireza Alemi, Davide Zoccolan

**Affiliations:** 1 Visual Neuroscience Lab, International School for Advanced Studies (SISSA), 34136 Trieste, Italy; 2 Center for Neuroscience, and Department of Neurobiology, Physiology, and Behavior, University of California, Davis, Davis, CA 95616, USA

**Keywords:** object recognition, perceptual strategy, invariance, artificial vs. biological vision, visual perception, rodents, convolutional neural networks

## Abstract

Despite their prominence as model systems of visual functions, it remains unclear whether rodents are capable of truly advanced processing of visual information. Here, we used a convolutional neural network (CNN) to measure the computational complexity required to account for rat object vision. We found that rat ability to discriminate objects despite scaling, translation, and rotation was well accounted for by the CNN mid-level layers. However, the tolerance displayed by rats to more severe image manipulations (occlusion and reduction of objects to outlines) was achieved by the network only in the final layers. Moreover, rats deployed perceptual strategies that were more invariant than those of the CNN, as they more consistently relied on the same set of diagnostic features across transformations. These results reveal an unexpected level of sophistication of rat object vision, while reinforcing the intuition that CNNs learn solutions that only marginally match those of biological visual systems.

## Highlights

• Rat object vision is modeled using a deep convolutional neural network (CNN)
• Half the CNN is needed to model rat tolerance to translation, scaling, and rotation
• The whole CNN is required to match rat performance with occluded and outlined objects
• The view-invariance of the rat discrimination strategy is unmatched by the CNN

## The bigger picture

Rodents have become very popular model systems to study the neuronal mechanisms underlying visual perception, yet it remains unclear how advanced their visual system really is. Here, we use a deep neural network to understand how computationally difficult it is for rats to succeed in different object-recognition tasks. We found that at least half of the network is required to match rat perception when objects undergo spatial transformations, such as translation and scaling. The whole network is needed instead to equate rat ability to recognize the target objects despite partial occlusion and reduction to outlines. In addition, rats process visual objects by using discrimination strategies that are more consistent across view changes than those of the network. These findings reassert the sophistication of rat vision while suggesting interesting adjustments to the architecture and visual diet of deep nets to bring their robustness and generalization power closer to their biological counterparts.

## Teaser

Muratore et al. modeled rat object vision using a deep convolutional neural network (CNN). They found that processing up to half of the CNN is needed to account for rat tolerance to translation, scaling, and rotation of target objects, while the whole network is required in the case of partial occlusion and reduction to outlines. Instead, the complexity and view-invariance of rat discrimination strategy remains unmatched by the CNN. These findings indicate that rats process visual objects by using advanced neuronal machinery.

## Introduction

The neuronal basis of visual object recognition has been the subject of intense study, with a particular focus on the ventral visual pathway—the visual cortical hierarchy that, in primates, is devoted to process object and shape information.^1^^,^^2^ Recent behavioral and neurophysiological evidence suggests that also rodents, and rats in particular, display non-trivial object-recognition abilities,^3^ thus offering a viable new model for studying the neuronal basis of object vision. Rats are able to recognize objects despite variations in pose, size, position, illumination, and visual cues.^4^^,^^5^^,^^6^^,^^7^ They can discriminate the content of natural movies^8^ and even categorize faces.^9^ In addition, rats display the same pattern of sensitivity to multi-point texture statistics that humans do,^10^ and they seem to process visual objects by relaying on non-trivial, multiple-feature perceptual strategies.^5^^,^^9^^,^^11^^,^^12^ These perceptual abilities are consistent with the hierarchical increase in the complexity and invariance of object representations found along the progression of extrastriate visual areas that run laterally to primary visual cortex (V1), pointing to this pathway as the rat homolog of the primate ventral stream.^7^^,^^13^^,^^14^^,^^15^^,^^16^^,^^17^

Despite the behavioral and neurophysiological evidence reviewed above, a limit of current visual perceptual studies in rodents is the relatively low number of visual stimuli they rely upon, which rarely exceeds 100 distinct object conditions (but see Alemi-Neissi et al.,^5^^,^^9^^,^^11^^,^^12^ Schnell et al.,^5^^,^^9^^,^^11^^,^^12^ Rosselli et al.,^5^^,^^9^^,^^11^^,^^12^ and Djurdjevic et al.^5^^,^^9^^,^^11^^,^^12^). By comparison, modern investigations of primate object vision benefit from high-throughput psychophysical techniques that allow testing at a much larger scale (i.e., order of thousands of images across hundreds of human participants).^18^^,^^19^ The limited size of stimulus sets used in rodent studies makes it difficult to fully exclude the possibility that the animals succeed in a given discrimination task by relying on trivial strategies based on detection of low-level visual cues. For instance, we have previously shown that, when objects are presented across a limited number of transformations (or views), differences of mean luminosity between the sets of views of two objects allow invariant encoding of object identity already at the level of V1 representations.^7^

One way to check whether the variety of stimulus conditions employed in an object-recognition task was large enough to engage high-order representations is to feed the same battery of conditions to a convolutional neural network (CNN) for image classification and then measure how well the task is solved across consecutive layers of the network. This approach relies on the fact that CNNs, with their hierarchical architecture and ability to learn complex representations, have proved to be the most successful artificial models for biological vision to date.^20^^,^^21^^,^^22^^,^^23^^,^^24^ In particular, several studies have found a hierarchical match between ventral stream areas and CNN layers in terms of the ability of neural activations in the latter to explain neuronal tuning in the former. That is, while activations in early CNN layers better predict responses to natural images in low-level visual cortical areas (e.g., V1), activations in deeper layers better account for neuronal tuning in higher stages of the ventral stream, such as the inferotemporal cortex (IT).^21^^,^^25^^,^^26^ This suggests that a similar approach could be used to assess the complexity of an object-recognition task by measuring at which depth of a CNN object representations successfully solve the task. Trivial discriminations would be solvable by representations in early layers, while more demanding tasks (e.g., in terms of invariance) would require the full extent of the neural architecture.

This procedure has been applied to assess the complexity of visual representations used by humans engaged in a rapid animal vs. non-animal categorization task.^27^ More recently, a first attempt to gauge the complexity of rat object vision using this approach was presented by Vinken and Op de Beeck.^28^ These authors found that rat classification accuracy reported in several object-recognition experiments^4^ was successfully modeled by very early layers of standard CNNs—more computational depth was required only when the task involved discrimination of natural movies.^8^ In a follow-up study,^29^ CNNs were used to devise a suitable image set to investigate the extent to which rats are capable of truly advanced object recognition. Although rat classification accuracy was best captured by activations in late convolutional layers of a CNN, the latter could only account for a small fraction of the variance of rat behavioral performance, and substantial differences emerged with the performance pattern of human participants tested in the same task.

Overall, these results suggest that former psychophysical studies may have overestimated the ability of rats to tolerate transformations in object appearance because the size and variety of the stimulus sets may not have been sufficiently large to rule out the deployment of low-level strategies. However, we believe that previous applications of CNNs to evaluate the complexity of rat vision lacked important details concerning the resolution and noise at the front end of the rat visual system, the way in which the animals viewed the stimuli in the absence of head fixation, and the cognitive constraints that affect perceptual decision making in rodents. To overcome these shortcomings, we developed an image pre-processing pipeline that explicitly models the constraints of rat vision (e.g., low visual acuity^30^^,^^31^) and the additional image variability (e.g., translations and in-plane rotations) induced by rat head movements during execution of the perceptual task.^32^ Furthermore, we went beyond a comparison between rats and CNNs in terms of absolute discrimination performances, which are not that meaningful, considering that the large lapse rates of rodent perceptual decisions^33^ are fully absent in CNN models. We focused instead on comparing how similarly recognition accuracy was modulated across object views in rats and CNN layers. In addition, we compared the visual strategies used by rats and CNNs to solve the same object-recognition tasks by applying to CNNs the image classification approaches that have been successfully used to infer rat visual perceptual templates in Alemi-Neissi et al.^5^^,^^12^ and Djurdjevic et al.^5^^,^^12^ This approach aligns with recent strides in the field of explainable AI^34^ and cognitive sciences^35^ that have highlighted how modern architectures for machine vision, despite their saturation of classification accuracies on challenging benchmarks, often exploit unintelligible visual strategies^36^ (e.g., features in the background) that substantially differ from those used by their biological counterpart.^37^^,^^38^

We found that, although mid-level layers of a standard CNN (VGG-16) are effective models of rat classification accuracy in tasks with moderate image variability, the full depth of the network, including the final, fully connected layers, is required to achieve the best match with rat performance on more challenging tasks, such as those involving partial occlusions or outline versions of the target objects. In addition, we found that rat visual perceptual strategies are more invariant than those used by the CNN. Rats make more consistent use of the same set of visual features, preserved across transformations, thus relying on a more parsimonious and generalizable collection of visual landmarks than the corresponding artificial counterpart. Interestingly, these findings are consistent with several of the discrepancies observed between humans and CNNs in terms of image processing (see discussion). This reasserts the sophistication of rat vision and the potential of rodents as model systems to investigate non-trivial visual computations. At the same time, it suggests possible adjustments to the architecture and visual diet of CNNs to improve their object awareness^39^ and their robustness to out-of-distribution image manipulations in which both primate and rodent visual systems appear to excel.

## Results

**Figure 1:**
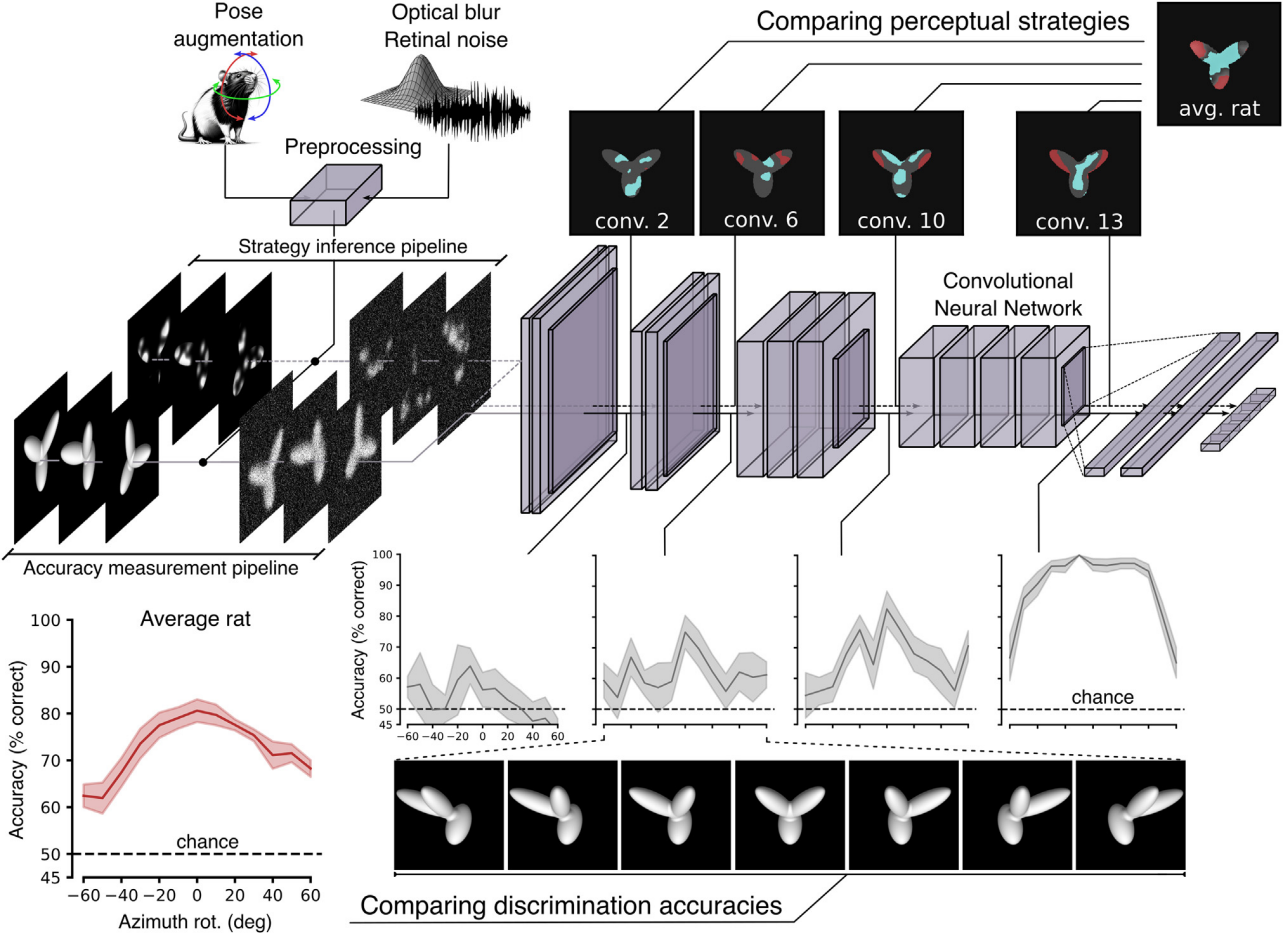
Comparing processing of visual objects in rats and CNNs Conceptual overview of the experiments we carried out on VGG-16 to assess the complexity of rat object vision. We investigated how consecutive layers of VGG-16 represent the visual stimuli that have been used in three previous studies of rat visual object recognition.^4^^,^^5^^,^^12^ Before being fed to the VGG-16 input layer, the visual objects were subjected to an image pre-processing stage to simulate the resolution/fidelity of rat spatial vision (by the addition of blur and noise) as well as the variations in object appearance produced by head movements (resulting in an augmented image set). Following the experimental design of the rat studies, visual objects were presented to the network in two different flavors. Unoccluded/unaltered images were used to measure how well object representations across VGG layers supported object discrimination (bottom branch: "accuracy measurement" pipeline). This allowed comparison of how well the pattern of rat discrimination accuracies across object transformations (e.g., azimuth rotations; red curve) aligned to the patterns of discrimination accuracies measured along the network (gray curves). Conversely, either partially occluded or structurally altered versions of the target objects were used to infer which visual strategies VGG representations afforded to support object discrimination (top branch: "strategy inference" pipeline). This allowed comparison of the set of diagnostic salient (red patches) and anti-salient (cyan patches) features used by the rats (rightmost saliency map) and by VGG (sequence of saliency maps along checkpoint layers) to succeed in the object-recognition task. For both pipelines, VGG layers were probed by training linear classifiers to predict the image labels based on the object representations they provided.

Our goal was to establish the extent to which rats are capable of advanced shape processing by comparing their performance patterns and perceptual strategies, as reported in three of our previous studies,^4^^,^^5^^,^^12^ with those afforded by object representations across the layers of VGG-16—a popular convolutional network^40^ that has been often used as a benchmark against which to compare biological vision (see, e.g., Güçlü and van Gerven,^26^^,^^28^^,^^41^^,^^42^^,^^43^^,^^44^^,^^45^ Vinken and Op de Beeck,^26^^,^^28^^,^^41^^,^^42^^,^^43^^,^^44^^,^^45^ Dobs et al.,^26^^,^^28^^,^^41^^,^^42^^,^^43^^,^^44^^,^^45^ Grossman et al.,^26^^,^^28^^,^^41^^,^^42^^,^^43^^,^^44^^,^^45^ Jaegle et al.,^26^^,^^28^^,^^41^^,^^42^^,^^43^^,^^44^^,^^45^ Rust and Mehrpour,^26^^,^^28^^,^^41^^,^^42^^,^^43^^,^^44^^,^^45^ and Higgins et al.^26^^,^^28^^,^^41^^,^^42^^,^^43^^,^^44^^,^^45^). This was achieved by feeding the CNN with the same object conditions employed in the three rat studies using the data-processing pipeline depicted in Figure 1.

Since our aim was to measure both the object discrimination accuracy and the underlying perceptual strategy, and since the latter was uncovered, in the rat studies, using two different classification image approaches (where the original objects were altered either by partial occlusion with random masks or by random structural variations), the visual objects fed to the network were either the unoccluded/unaltered objects ("accuracy measurement" pipeline in Figure 1) or their occluded/structurally altered versions ("strategy inference" pipeline). In both cases, before being fed to the VGG input layer, the images of the visual objects were subjected to a pre-processing step to augment the original stimulus dataset in such a way as to: (1) better match it to the low resolution of rat spatial vision (i.e., by adding blur and noise); and (2) simulate the additional variations in object appearance induced by head movements.^32^

We then followed an approach that is similar to the one adopted by previous studies comparing CNNs’ with humans’ or rats’ proficiency in object-recognition tasks^27^^,^^28^ (see also discussion). We used linear classifiers to read out the identity of the objects fed to VGG-16, based on their representations in each layer of the network. This method allows probing the extent to which object representations at a given stage of a processing hierarchy are sufficiently untangled to support transformation-tolerant recognition by a simple, linear readout scheme.^2^^,^^46^ By systematically and independently applying a linear decoder to each layer of VGG-16, we could thus scan the depth of the network, pursuing two complementary goals: (1) to look for the processing stages yielding the patterns of discrimination accuracy that were most consistent with those measured for the rats ("accuracy measurement" pipeline: see the bottom branch of Figure 1); or (2) to infer the visual discrimination strategies supported at each stage of the network and compare them to the known perceptual templates deployed by the rats ("strategy inference" pipeline: see the top branch of Figure 1).

More specifically, we trained a linear support vector machine (SVM) per layer on the object classification task using, as feature vectors, the activations of a random subpopulation of units for the training views of the two objects. In any given experiment, the size of the subpopulations sampled from each convolutional layer was held constant to control for the possible impact of the dimensionality of the representational space on classification accuracy. In addition, since the layers widely differed in the total number of units, we further controlled for potential population undersampling by repeating each test with an increasing (logarithmically spaced) number of units (from $10^{3}$ to $10^{5}$), capping them to the total size of a layer when needed (i.e., in the fully connected layers). Finally, each trained SVM was tested for its ability to correctly classify the activations produced by the held-out test images, and both the train and test accuracies were recorded.

### Image-augmentation pipeline to account for rat visual acuity and head movements

**Figure 2:**
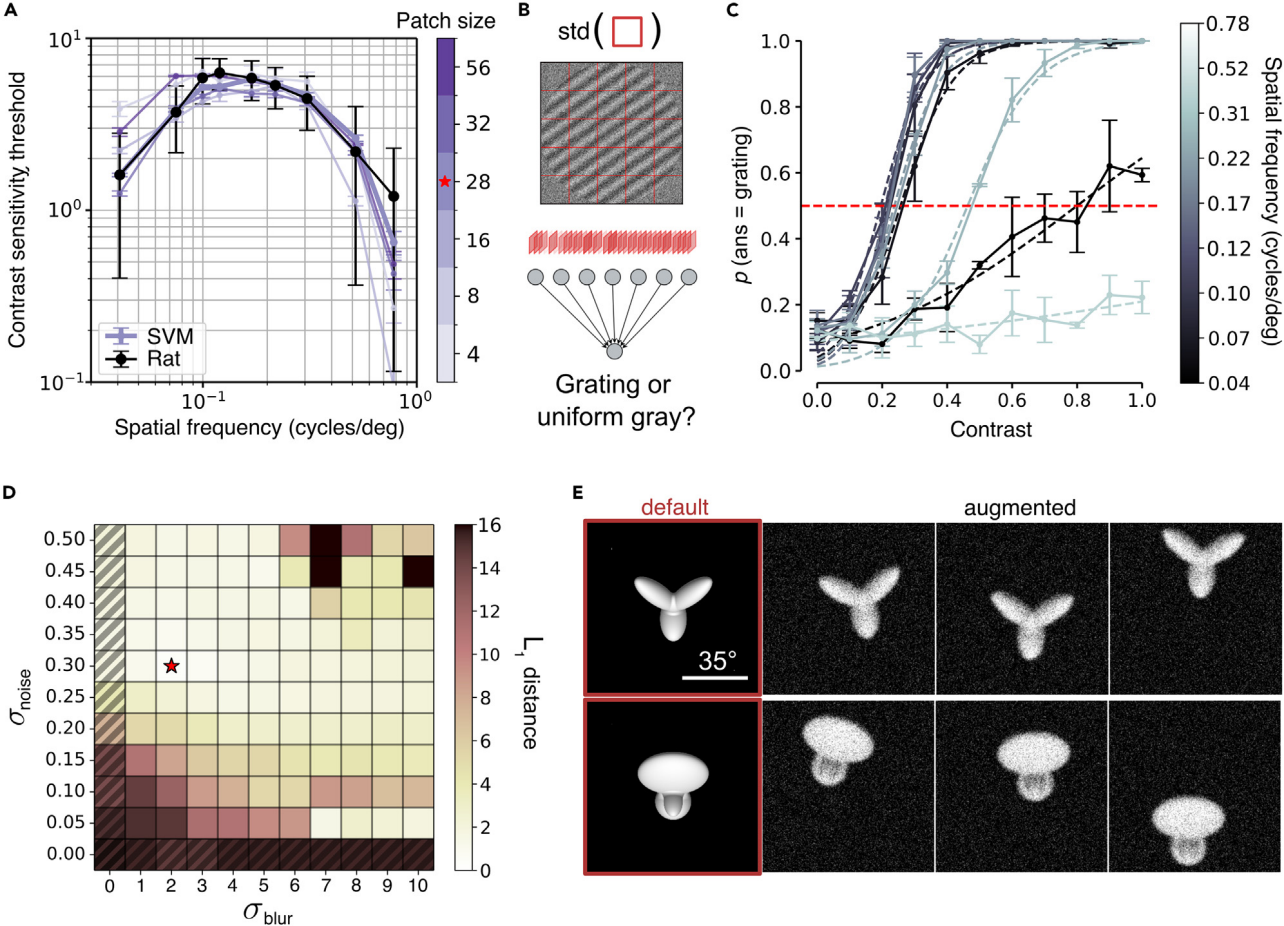
Image-augmentation pipeline to account for rat visual acuity and head movements (A) The contrast sensitivity function (CSF) measured for rats by Keller et al.^30^ (black dots/line) is shown along the CSFs (purple curves) obtained for a simulated observer, measuring the contrast of input images in small patches with different sizes (as illustrated in B). The curves all refer to the same combination of image blur and noise marked by the red star in (D). The red star on the color bar marks the patch size yielding the CSF with the best fit to the rat CSF. (B) Schematic representation of the grating-detection task used to obtain the CSFs of the simulated observer. The task consisted in detecting whether the input image was a grating, as opposed to a uniform gray square, by relying on the contrast measured over a grid of patches within the image (red squares). The images were subjected to various degrees of Gaussian blur and additive noise. The simulated observer consisted in a linear classifier, fed with the contrast values measured across the image patches. (C) Psychometric-like functions, showing the probability of the simulated observer to detect an input grating (with a given spatial frequency) as a function of its contrast. The contrast level at which a given psychometric crossed the p=0.5 value was taken as the contrast threshold for that particular spatial frequency. Such thresholds were used to obtain the CSFs, as explained in methods. The curves shown here were obtained for the combination of parameters marked by the red star in (D) and path size =32. Error bars denote the SD over 3 repetitions. (D) $L_{1}$ distance between the rat CSF and the CSFs of the simulated observer that were obtained over a combination of $\sigma_{\text{noise}}$ and $\sigma_{\text{blur}}$ parameters for patch size =28. The red star marks the combination yielding the best fit (same for all tested patch sizes). (E) Outcome of the full augmentation pipeline applied to the default views of the objects used in Zoccolan et al. and Alemi-Neissi et al.^4^^,^^5^ For each of the original objects (highlighted in red), three augmented views are provided, resulting from adding the blur and noise levels yielding the best fit with rat CSF and from applying the expected vertical/horizontal shifts and in-plane rotations produced by head movements.

A fair comparison between rat visual perception and CNN models requires the latter to incorporate some key ecological constraints on the native resolution of the rat visual system. Rats have low visual acuity, being able to resolve at most a spatial frequency (SF) of 1 cycle/deg.^30^^,^^31^^,^^47^ This imposes a filter on the quality of the visual information that the front end of the rat visual system conveys to the rest of the brain. Spatial acuity is typically quantified via the contrast sensitivity function (CSF).^30^ The CSF is inversely related to the minimal amount of contrast that is required for successful detection of a sinusoidal grating at a given spatial frequency (see methods). In the case of Long-Evans rats, the curve peaks at 0.1 cycles/deg and drops to zero for SFs smaller than 0.04 cycles/deg and higher than 1 cycle/deg (black curve in Figure 2A). This shape results from the interplay of several factors, such as the quality of the optics of the eye, the density of cones and rods in the retina, and the level of neuronal noise in the photoreceptors and the other retinal cell types.^48^^,^^49^^,^^50^ All these factors contribute to determine the quality of the retinal image, which needs to be taken into account in the assessment of rat pattern vision. Since developing an anatomical and biophysical model of the encoding of retinal images would be a daunting task, also because of the paucity of available data, we resorted to a functional modeling approach. Considering the CSF as the aggregate functional measurement of the sharpness/resolution and fidelity of rat spatial vision, we searched for the combination of image blurring and noise that best reproduced the rat CSF.

To simulate the grating detection task used to measure the CSF, we trained a linear SVM to discriminate noisy and blurred sinusoidal gratings from noisy, uniform mid-gray images. More specifically, we divided the simulated stimulus display in a grid of squared patches, we measured the image contrast within a patch as the standard deviation of its pixel intensity values and took the resulting set of patch contrasts as the feature vector to be fed to the SVM (Figure 2B). Multiple samples (5,000) for each class of stimuli (gratings and mid-gray images) were produced by random variations of the additive noise pattern applied to the images and, in the case of the gratings, by random variations in phase and orientation.

This pipeline simulated an observer that tries to detect the presence of a grating based on the luminance contrast extracted from the stimulus display. This allowed measuring the detection performance of the simulated observer as a function of image contrast and spatial frequency, thus obtaining psychometric functions from which we could estimate the contrast thresholds to detect the gratings at each SF (Figure 2C). Based on these thresholds, we could compute the CSF for the simulated observer and compare it to the rat experimental CSF measured by Keller and colleagues^30^ and reported in Figure 2A (black curve). To estimate the blur and noise levels that yielded the closest match (i.e., lowest $L_{1}$ distance) between simulated and experimental CSFs, we performed a grid search over the Gaussian blur intensity and the Gaussian noise applied to the input images (quantified via their variance $\sigma_{\text{blur}}^{2}$ and $\sigma_{\text{noise}}^{2}$, respectively), as well as over the patch size *p* (Figure 2D). The resulting $L_{1}$ landscape over the $\left(\sigma_{\text{blur}}^{2},\sigma_{\text{noise}}^{2}\right)$ plane featured a flat valley (light cells) with the absolute minimum (marked by the star) yielding a very precise fit with the experimental CFS regardless of patch size (Figure 2A; compare the purple curves to the black curve). Thus, with a moderate level of blur and noise, we could functionally and accurately simulate the quality of the images encoded by the rat retina and use this pre-processing step in all our tests with VGG-16.

A second pre-processing stage was also applied to take into account the fact that all perceptual studies of rat object vision to date have been carried out without head fixation and gaze control.^3^ In the experiments performed by our group, some level of body restraint has been achieved by requiring the animals to protrude the head through a narrow viewing hole placed in front of the stimulus display.^4^^,^^5^^,^^10^^,^^11^^,^^12^^,^^51^^,^^52^ This allows for a good control over viewing distance and, therefore, stimulus size, but does not fully prevent head movements. In particular, we found that head orientation at the time of stimulus presentation is not reproducible across trials: pitch, roll, and yaw rotations vary over a span of about 60°, 35°, and 20°, respectively.^32^ Obviously, these trial-by-trial variations in head orientation induce additional transformations on the images of the visual stimuli over the rat retina in addition to the image variability designed by the experimenters. Specifically, pitch and yaw rotations translate into vertical and horizontal shifts, while roll rotations produce in-plane rotations. With knowledge of the geometry of the experimental rig and basic trigonometry, one can easily compute the corresponding image transformations (see methods). These can be considered as augmented versions of the training and test images used to probe object recognition in rats. As an example, in Figure 2E we have reported the effects of the described augmentation on the images of the original stimuli used in Zoccolan et al.^4^^,^^5^ and Alemi-Neissi et al.^4^^,^^5^ The default views of the objects used in those studies are shown on the left (highlighted by the red frames), while a few instances of augmented versions (incorporating also the blur and noise produced by the pre-processing step described above) are displayed on the right.

Since, in a given trial, head orientation along the three rotation axes was random (within the ranges reported above), to properly simulate the actual level of image variation experienced by the rats during the training/testing in our perceptual tasks, we implemented the following pipeline. For each visual image that had to be fed to the CNN, we first randomly sampled the values of pitch, roll, and yaw within the allowed ranges, after which we translated them into the corresponding vertical/horizontal shifts and in-plane rotations of the image. After this augmentation step was completed, we added the blur and random noise perturbations and finally fed the image to the network. For brevity, in what follows, we will refer to this whole pre-processing pipeline (including augmentation, blurring, and noise) simply as “augmentation.” The pipeline was consistently applied to all the images fed to the CNN that are described in our study, as illustrated in Figure 1.

### The best match with rat tolerance to size changes and in-depth rotations is achieved in VGG-16 middle layers

**Figure 3:**
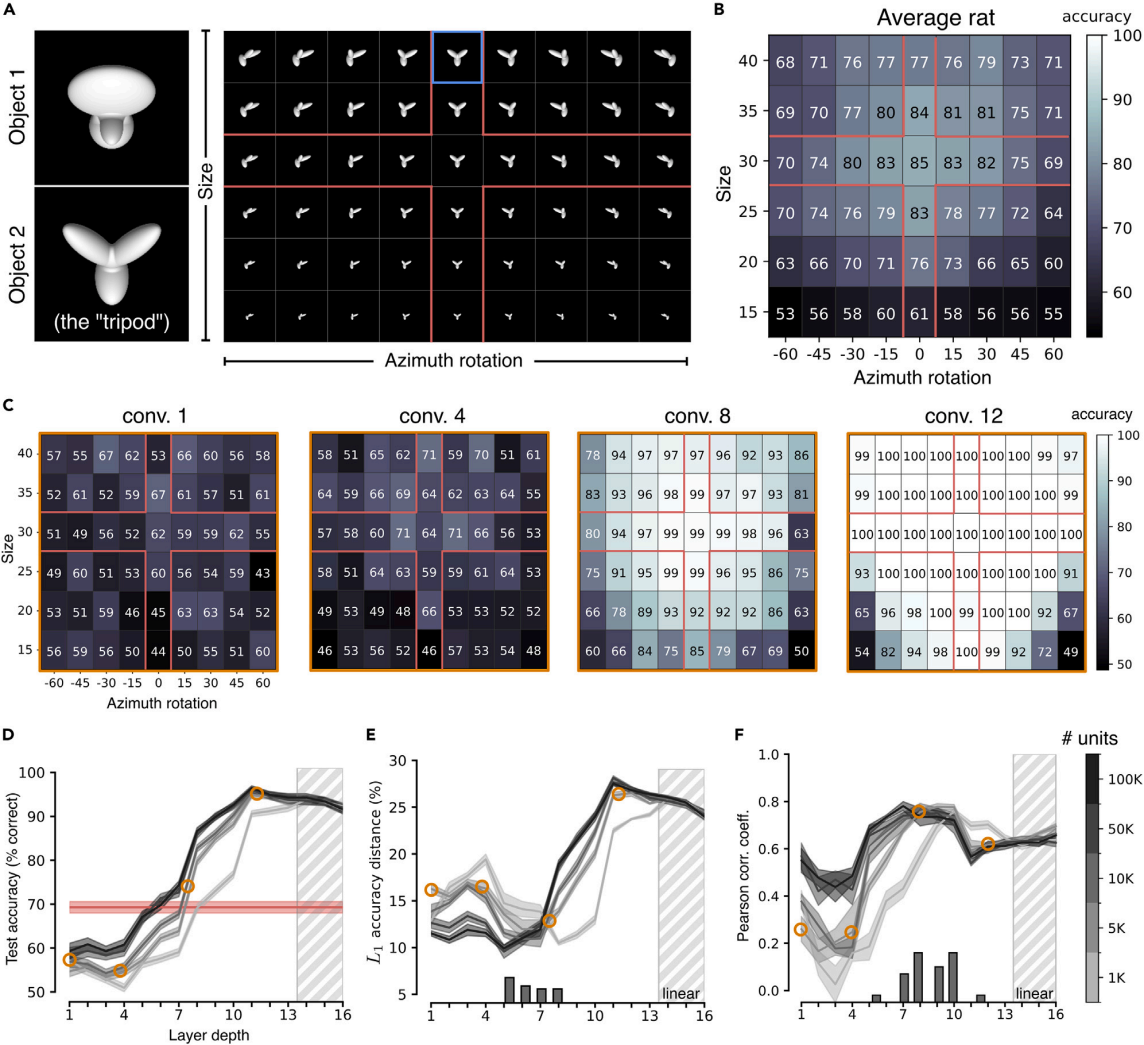
VGG-16 mid-level layers match the pattern of rat discrimination accuracy across size changes and in-depth rotations (A) The stimulus set originally used by Zoccolan et al.^4^ Two objects (default views shown on the left) were presented across a combination of size and azimuth in-depth rotations (shown on the right for one object). The blue frame indicates the view used for the initial training of the animals. The red cross indicates the additional views the rats were trained with before being tested with the off-cross views. (B) Group average performance of the rats across the set of object transformations shown in A. Adapted from Zoccolan et al.^4^ (C) Discrimination performances achieved by training SVM classifiers with the activations of VGG-16 units to the on-cross object views and testing them with the activations to the off-cross views of the stimulus matrix shown in (A). The four performance matrices refer to activations sampled from progressively deeper convolutional layers of the network. (D) Test accuracies (i.e., performances measured on the off-cross views of A) of the SVM classifiers achieved across the depth of VGG-16 (gray curves). Shaded areas are SEM over five different classification runs (see methods). The shades of gray indicate the number of units that were sampled from each layer and fed to the SVMs (see color bar on the right of F). Rat average test accuracy is reported in red. The orange circles highlight those layers whose accuracy patterns have been displayed in (C). (E) $L_{1}$ distance between the pattern of accuracy of the rats across the whole stimulus matrix (i.e., both on- and off-cross cells in B) and the patterns of accuracy obtained across VGG-16 layers. The histogram in the bottom reports the occurrence of the minima of the $L_{1}$ distance across classification runs and sizes of the populations fed to the SVMs. Orange circles and shaded areas as in (D). (F) Same analysis as in (E), but carried out by computing the Pearson correlation coefficient instead of the $L_{1}$ distance.

As a first assessment of the complexity of rat object vision, we considered the experiment originally presented by Zoccolan and colleagues,^4^ who tested the ability of rats to discriminate two visual objects despite variations in size and in-depth rotations (the two visual objects are shown in Figure 3A, left; the full set of transformations applied to one of the objects is shown in Figure 3A, right). The rats were initially trained to discriminate two specific (“frontal”) views of the objects (those shown in Figure 3A, left), presented at 40° of visual angle (blue frame in Figure 3A, right). They were then trained on scaled versions of the frontal views (down to 15° of visual angle) and on in-depth, azimuth rotations of the objects (from −60° to +60°) shown at 30° of visual angle (these further training conditions are indicated by the red cross in Figure 3A, right). Finally, the rats were tested on unseen combinations of size and azimuth transformations (off-cross conditions in Figure 3A, right). The animals achieved above-chance discrimination accuracy across virtually all tested conditions, although the performance decreased as a function of the magnitude of the transformation (Figure 3B).

To check the extent to which this performance pattern is consistent with advanced processing of object information, we administered the same task to VGG-16, following the accuracy measurement pipeline illustrated in Figure 1. We used VGG-16 pre-trained to achieve high classification performances on ImageNet,^53^ which ensured that populations of units across progressively deeper stages of the network represented increasingly complex combinations of features found in natural images in an increasingly transformation-tolerant way.^54^^,^^55^^,^^56^ We fed the network with thousands of randomly augmented versions (see previous section) of the two objects used in the rat experiment sampled from the whole pool of transformations (i.e., both on- and off-cross views in Figure 3A, right). Following the approach described in Figure 1, we trained linear SVMs on the object discrimination task using train examples only (i.e., on-cross images in Figure 3A, right), and we tested the classifiers on held-out views (i.e., off-cross images), looking for the processing stages that yielded the pattern of discrimination accuracy that was most consistent with the one measured for the rats (shown in Figure 3B).

Figure 3C reports the patterns of discrimination accuracies of the SVM classifiers over the combination of size and azimuth transformations that we measured across progressively deeper checkpoint layers of VGG-16. A qualitative inspection of these patterns shows a progression from near-chance performances in initial layers toward saturating, near-perfect performances in the final ones. This trend was quantified in Figure 3D, which reports the average performance (gray lines) across all object views included in the test set (i.e., off-cross cells in Figure 3A, right) along with the average performance measured for the rat on those same conditions (red line). Discrimination accuracy started close to chance and remained such until layer 4, where it started to increase sharply, crossing rat performance between layers 6 and 8, depending on the size of the subpopulations of units fed to the SVMs. Larger subpopulations (darker curves) yielded higher performances, although the difference was mainly appreciable when the number of units increased from 1K to 5K, while further expansions until 100K only added a marginal gain. Performance saturated near to 90% correct in the deepest layers.

A finer-grain comparison between the magnitude of rat and VGG performances was obtained by computing the $L_{1}$ distance between the performance matrix of the rats (i.e., the data shown in Figure 3B) and the performance matrices obtained across consecutive layers of the network (like the examples shown in Figure 3C). The smallest distance was observed between layers 5 and 8 (Figure 3E), as shown by the distribution of the $L_{1}$ minima obtained across five different classification runs for each subpopulation size (histogram at the bottom of Figure 3E).

Overall, according to these analyses, it takes between six and eight convolutional layers of a powerful CNN architecture to match rat accuracy on the object-recognition task used in Zoccolan et al.^4^ This shows that dealing with the level of image variability imposed by the task is not trivial and requires substantial processing. This conclusion is, by itself, in contrast with the one of Vinken and Op de Beeck,^28^ who found that the VGG-16 first layer already surpassed rat performance on the task (see discussion). More importantly, as already mentioned in the introduction, we believe that comparing the rat visual system to CNNs in terms of absolute performances is not very meaningful in the first place. The two systems are too different, not only at the level of the basic architecture (i.e., number of processing stages, number of units, and so forth) but even in terms of perceptual decision strategies. For instance, rats display large lapse rates, i.e., constant rate of errors, even on “easy” stimuli (where performance should be perfect), which do not reflect an inability to correctly recognize the stimuli but, rather, the deployment of exploratory strategies.^33^ These lapses impose a cap on the maximal accuracy a rat can reach on a given task, thus leading to a systematic underestimation of the actual perceptual discriminability of the stimuli.

In the light of these considerations, we also carried out a different sort of comparison, measuring the Pearson correlation coefficient between the patterns of discrimination accuracies obtained for the rats and each VGG layer across the transformation matrix. Being scale and shift invariant, Pearson correlation allows assessing the extent to which rat and VGG performances are similarly modulated by the combinations of size and azimuth variations, regardless of the magnitude of the performance. As shown in Figure 3F, Pearson correlations steadily raised across the depth of VGG, reaching a flat peak between layers 7 and 10 (see the histogram of maximal correlations across classification runs and subpopulation sizes at the bottom of Figure 3F). The magnitude of the correlations at the peaks was substantial (about 0.8), thus confirming the visual impression of a very tight match between the classification performance landscapes obtained for the rats and for middle layers of VGG-16 (compare Figure 3B to the third matrix in Figure 3C). This result indicates that, when absolute performance magnitudes are left aside, it takes at least half the computational depth of VGG-16 to closely match rat sensitivity to the variations in object appearance tested by Zoccolan and colleagues.^4^

### The best match with rat robustness to partial occlusion is achieved in VGG-16 deepest layers

**Figure 4:**
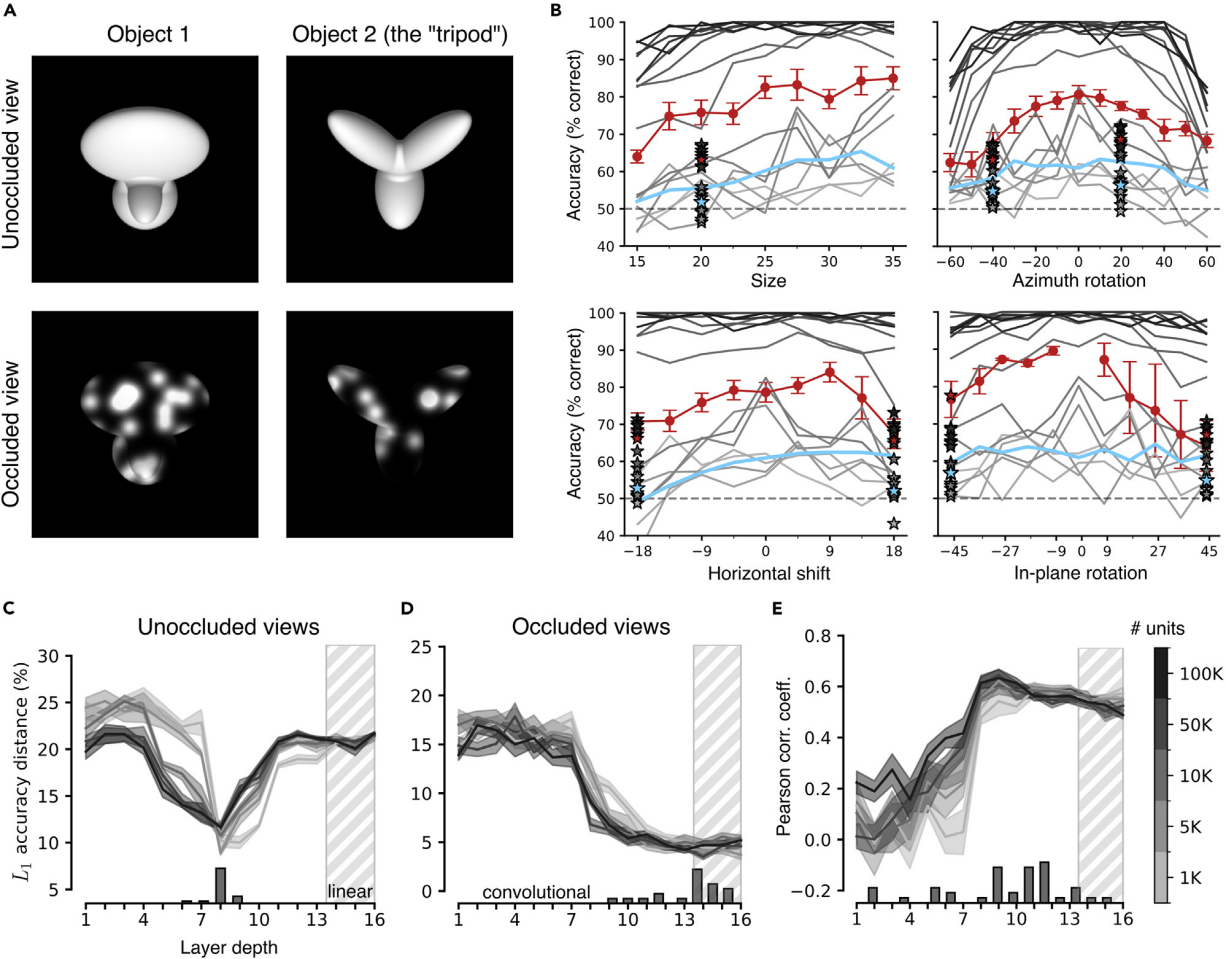
VGG-16 deepest layers match rat tolerance to partial occlusion (A) The default views of the two objects used by Alemi-Neissi et al.^5^ (top) are shown along two examples of partially occluded views (bottom). (B) Classification accuracy of linear SVMs trained to discriminate the two visual objects shown in (A) based on the representations provided by the activations of VGG-16 units across progressively deeper layers (100K units were randomly sampled from each layer). Discrimination accuracy was measured along four different axes of image variation: size, azimuth rotation, horizontal shift, and in-plane rotation. The performances of linear SVMs directly trained on the pixel-level representations are shown in cyan. The gray curves report the accuracy measured on unoccluded images across five repetitions of the classification procedure (see methods). The gray stars indicate the accuracy achieved by the SVMs on a few selected object views that were partially occluded (as shown in A, bottom). The shades of gray (from light to dark) code for layer depth (from early to deep). The group average performances achieved by the rats with the unoccluded and occluded object views are reported, respectively, by the red curves and stars. Error bars are SEMs. (C) $L_{1}$ distance between the pattern of accuracy of the rats across the whole set of unoccluded images (i.e., all red curves in B) and the patterns of accuracy achieved by the SVMs on the same images across VGG-16 layers. The shades of gray indicate the number of units that were sampled from each layer and fed to the SVMs (see color bar on the right of E). The shaded areas are SEM across five classification runs. The histogram reports the occurrence of the minima of the $L_{1}$ distance across classification runs and sizes of the populations fed to the SVMs. (D) Same as in (C), but with the $L_{1}$ distance computed between the accuracy patterns of the rats and the SVMs in the case of the partially occluded images. (E) Pearson correlation coefficient between the accuracy patterns achieved by the rats and the SVMs across the entire stimulus set (i.e., pooling unoccluded and occluded views) as a function of VGG-16 depth. Shaded areas are SEM as in (C). The histogram shows the distribution of the peaks of the correlation coefficient across classification runs and population sizes.

A limitation of the image set used by Zoccolan and colleagues^4^ (Figure 3A) is that it included only two kinds of object transformations: size changes and in-depth azimuth rotations. Other studies have probed the tolerance of rat object vision with a wider variety of transformations. In particular, Alemi-Neissi and colleagues^5^ probed rat vision with the same objects used by Zoccolan’s group but subjected also to horizontal translations and in-plane rotations, in addition to size and azimuth variations. Moreover, rats were also exposed to versions of the objects that were partially occluded by opaque masks punctured by randomly located transparent openings, or “bubbles” (examples of unoccluded and occluded conditions for the two objects are illustrated in Figure 4A). Overall, this offered the opportunity to investigate the similarity between rat and VGG-16 invariant recognition in the case of a wider, more challenging pool of image transformations.

The test performed with VGG-16 followed the same procedure as the one described in the previous section, measuring the accuracy of linear SVMs to classify the two objects (presented across the full set of transformations used by Alemi-Neissi and colleagues^5^), based on the activations of subpopulations of units sampled from each layer of the network. Also in this case, before being fed to the CNN, each image was first pre-processed through the augmentation pipeline. Given the randomness of the augmentation process and of the bubble masks, this resulted in two independent sets with thousands of images each: one to be used for training the SVM classifiers and another to test their performances (see methods).

The gray curves in Figure 4B show the test accuracies of the SVMs across the depth of the network (encoded by the gray shading) as a function of the magnitude of the four transformations probed by Alemi-Neissi and colleagues.^5^ Accuracy in the very initial layers (light gray) was close to chance, poorly modulated over the transformation axes, and even lower than the performance achieved by training an SVM to decode object identity directly from the pixel representations of the object views (cyan curves). It then progressively increased along the depth of the network, reaching, in middle layers (mid gray), an overall magnitude that was similar to the one measured for the rats (red curves). Concomitantly, the accuracy curves became increasingly closer to those of the rats also in terms of shape, eventually displaying similar transformation dependence in middle layers. Accuracy kept increasing with the depth of the network, substantially surpassing rat performance and approaching 100% correct in the fully connected layers (darkest gray). At the same time, the shape of the accuracy curves remained somewhat consistent with that observed for the rat curves even in deep convolutional layers.

Figure 4B also reports a comparison of rat and SVM performances (red and gray stars, respectively) on the object views that, in the study of Alemi-Neissi and colleagues,^5^ were partially occluded by the bubble masks. Rat recognition was quite robust to such manipulations, suffering only a minor drop of classification accuracy (the red stars are either at the same height or just below the corresponding dots on the red curves, reporting the performance on the unoccluded views). By contrast, the SVM classifiers, built over the representations provided by VGG layers, were afflicted by substantial performance losses (again, the accuracy yielded by the very initial layers was lower than that afforded by the pixel-level representation; compare light-gray and cyan stars). Only the deepest layers (darkest stars) afforded performances comparable with those of the rats.

The trends reported in Figure 4B refer to experiments where 100K VGG units were sampled per layer, but these results were very robust across subpopulation sizes and multiple runs of the classification procedure. This can be appreciated by inspecting the curves showing the $L_{1}$ distances between the performance patterns observed for the rats and the SVM classifiers across VGG layers. In the case of the unoccluded object views, the smallest $L_{1}$ distance was consistently reached in the middle of the network, with a sharp minimum in layer 8 regardless of population size (Figure 4C). By contrast, for the partially occluded views, the $L_{1}$ distance remained large and remarkably stable across the first half of the network, dropping sharply around layer 8 but then still decreasing until the very last layers (Figure 4D). As a result, most $L_{1}$ minima were concentrated in the final, fully connected layers.

As done in the previous section, we also computed the Pearson correlation coefficient between the accuracy patterns measured for the rats and the SVM classifiers across all tested image transformations (i.e., both unoccluded and occluded views). This similarity metric increased steadily until the middle layer, reaching a stable plateau in the second half of the network, with most minima concentrated between layers 9 and 12 (Figure 4E).

Overall, comparing these results with those of the previous section further reaffirms the sophistication of rat object vision. In fact, when visual objects underwent a richer variety of image transformations, compared to those tested by Zoccolan and colleagues,^4^ the pattern of rat discrimination performances could only be captured by deep convolutional layers of VGG-16. Most strikingly, the robustness of rat recognition to severe occlusion of the object views was only matched by the network in the final, fully connected layers.

### Comparing visual processing strategies in rats and CNNs

When using a convolutional neural network to model biological vision, comparing the two systems in terms of their discrimination accuracy only provides a first-order assessment of the model’s ability to capture the processing performed by the biological system. In fact, the same classification choices can in principle be supported by very different visual strategies, i.e., by very different sets of diagnostic features in the input images.^35^ For instance, recent studies have shown that deep neural networks, despite their high classification accuracies, often employ image-processing strategies that are substantially different from those used by humans.^36^ This imposes a limit on the ability of neuronal network models to account for the tuning of ventral stream neurons—a limit that can be overcome by enforcing an alignment with human perceptual strategies.^37^^,^^38^

Inspired by these previous studies, we extended our assessment of rat object vision using VGG-16 by including a comparison at the level of visual processing strategies. This was possible thanks to previous work of our group, in which two different classification image approaches have been applied to uncover the diagnostic features used by rats to discriminate visual objects.^5^^,^^11^^,^^12^ These approaches are based on: (1) the already mentioned occlusion of object views by random bubble masks (see previous section and Figure 4A)^5^; and (2) the presentation of randomly morphed variants of a previously learned target object.^12^ By properly processing rat responses to these altered object conditions, both approaches allowed estimation of the perceptual templates deployed by rats to recognize visual objects across various image transformations. In our tests with VGG-16, we adapted these procedures to infer the visual features underlying the choices of the SVM classifiers that were built over the representations provided by the network’s layers. This allowed comparison of the rats and the CNN in terms of the similarity and generalization across transformations of their perceptual strategies.

### Rat image-processing strategy is more view invariant than VGG-16 strategy and more consistent with that of an ideal observer model

Alemi-Neissi and colleagues^5^ collected rat responses to partially occluded versions of previously learned visual objects (Figure 4A). This allowed application of a classification image approach known as the bubbles method, originally developed to infer the object classification strategies of humans^57^ and later applied also to other species such as pigeons,^58^^,^^59^ monkeys,^60^^,^^61^ and, in the work of our group and others, rats.^5^^,^^11^^,^^62^

Intuitively, partial occlusion of visual objects impairs their processing, but the extent to which recognition becomes more difficult depends on which parts of an object are masked. If visual features that are critical to identify the object are erased, this will likely lead to misclassification. Conversely, occluding image features that are not very diagnostic of object identity will not alter classification. As originally proposed by Gosselin and Schyns,^57^ critical (or salient) features can be inferred by measuring the correlation between the random bubble masks and the behavioral responses of the observer to the corresponding partially occluded object conditions (see methods for details).

In our tests, we adapted this experimental procedure to CNNs by feeding VGG-16 with partially occluded object views and recording how the linear SVMs, trained on layer-specific activations, classified these images (see the "strategy inference" pipeline in the top branch of Figure 1). As already done for the analysis shown in Figure 4, the SVMs were trained using thousands of unmasked and randomly masked object views, equally divided in these two categories (see methods). This was done to closely match the task that had been administered to the rats, who received feedback about the correctness of their responses to both unoccluded and occluded images.^5^ In addition, early tests revealed that inclusion of masked objects in the training pool was essential to avoid degenerate classification performances on these conditions (i.e., perfect classification of one object and concomitant “perfect” misclassification of the other one). This is consistent with the poor generalization afforded by VGG representations to masked images, as already shown in Figure 4B. After training, each classifier was exposed to 3,000 partially occluded images for each of the object views that had been tested with bubble masks in the rat experiment^5^ (i.e., the seven views indicated by the stars in Figure 4B plus the default view, yielding a total of 16 views, considering the two objects).

**Figure 5:**
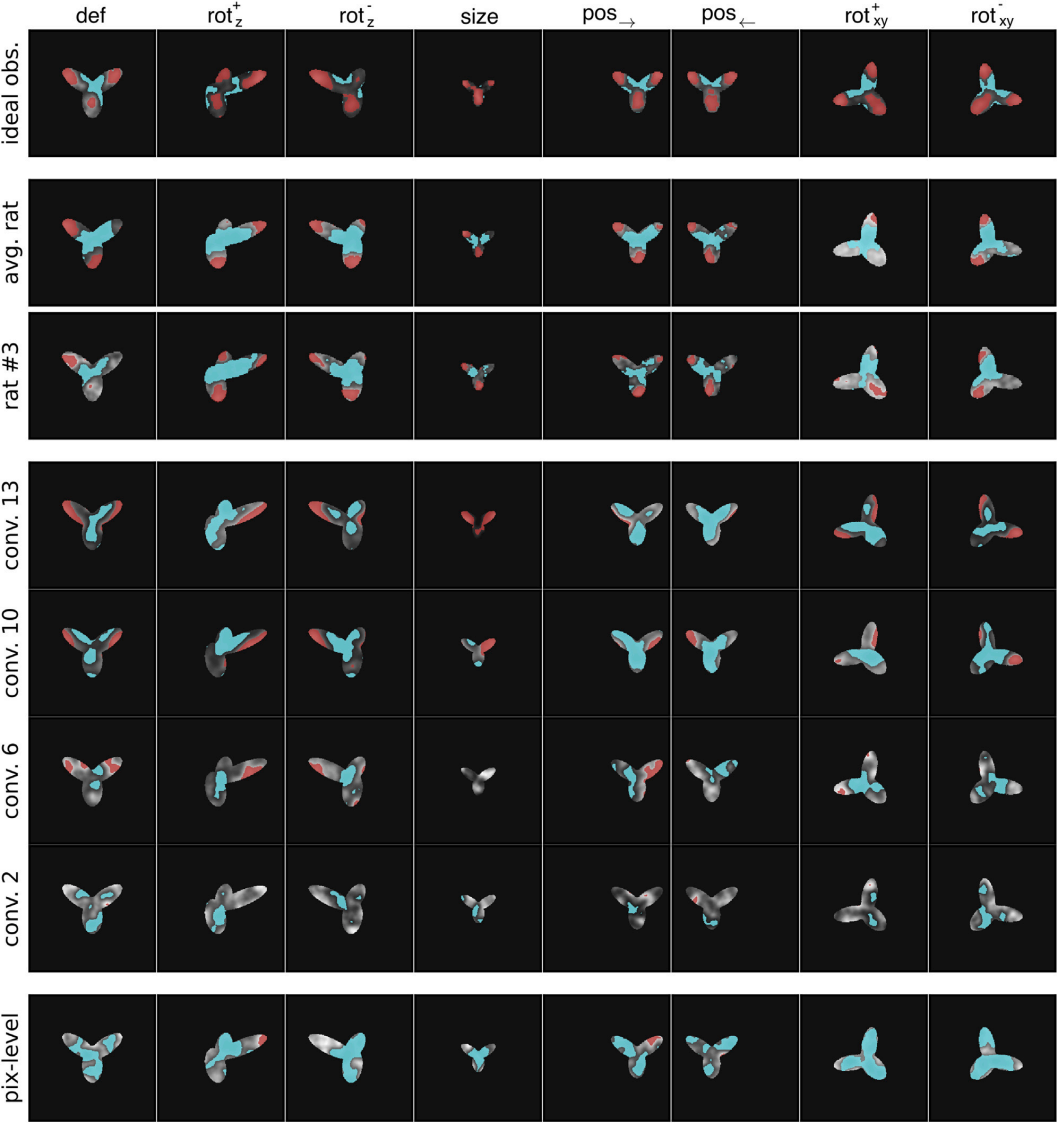
Comparing visual strategies among rats, VGG-16 layers, the pixel-based representation, and an ideal observer model The image classification approach known as the bubbles method was applied to uncover the saliency maps used to recognize the tripod object by four different “perceptual” systems: (1) an ideal observer model (described in the main text; first row); (2) the rats, with the group average maps shown in the second row and the maps of an example animal (rat #3; see Alemi-Neissi et al.^5^) shown in the third row; (3) linear SVMs applied to VGG-16 representations of the object in four checkpoint layers (rows 4–7); and (4) linear SVMs applied to the object’s pixel-based representation. In each map, the lightness of the grayscale indicates how well the visibility of a pixel correlated with the correct classification of the object (see methods). The red and cyan colors indicates pixels that were, respectively, significantly correlated and anti-correlated with the correctness of the classification (p<0.05; permutation test). These pixels form, respectively, significantly salient and anti-salient regions.

Figure 5 shows, for each of the eight views of the “tripod” object, the group average saliency maps that were obtained for the rats by Alemi-Neissi and colleagues^5^ (second row) as well as those obtained for one example animal (third row). The figure also reports the saliency maps that we computed for the SVM classifiers trained on VGG units’ activations in a few convolutional layers (rows 4–7) and on the pixel representations (last row). Following our rat study,^5^ the figure also shows the saliency maps obtained for an ideal-observer model (top row)—i.e., a classifier that had stored in memory, as templates, the eight views each object could take and that performed a template-matching operation (dot product) between each occluded input image and these 16 templates (assigning the object label based on the template that yielded the best match; see Alemi-Neissi and colleagues^5^ for details). This simulated observer was “ideal” in the sense that had complete knowledge of the variability of object appearances that was present in the stimulus set and could thus solve the invariant task optimally. In Figure 5, the intensity of the maps is rendered in shades of gray, where light and dark pixels indicate, respectively, strong correlation and anti-correlation between the visibility of the pixels through the masks and correct classification of the object. That is, light and dark shades indicate portions of the object views that are, respectively, diagnostic (or salient) and anti-diagnostic (or anti-salient) with respect to object identity. The red and cyan patches highlight those object regions that were significantly salient and anti-salient according to a permutation test (p<0.05; methods).

As already illustrated in Alemi-Neissi and colleagues,^5^ the saliency maps of both the average and the example rats featured multiple salient regions, located at the tips of the lobes of the tripod, as well as a central anti-salient area, located at the lobes’ intersection. Importantly, the relative position and size of the salient features was well preserved across views. By comparison, the saliency maps yielded by the representations in the pixel space and in VGG early layers were much less sharp, failing, in most views, to reach significance (i.e., little or no red patches are visible in the maps in the bottom rows of the figure). Also, the location of the salient regions (light gray) varied considerably from view to view. Stronger salient features emerged in late convolutional layers, although their shape was only partially consistent with that of the features in the rat maps, being located mostly at the edges rather than at the tips of the lobes. In general, regardless of the layer considered, the similarity between rat and VGG (or pixel) maps appeared to be low. By contrast, as already reported by Alemi-Neissi and colleagues,^5^ both the average and individual rat saliency maps displayed a remarkable similarity with those of the ideal observer model.

**Figure 6:**
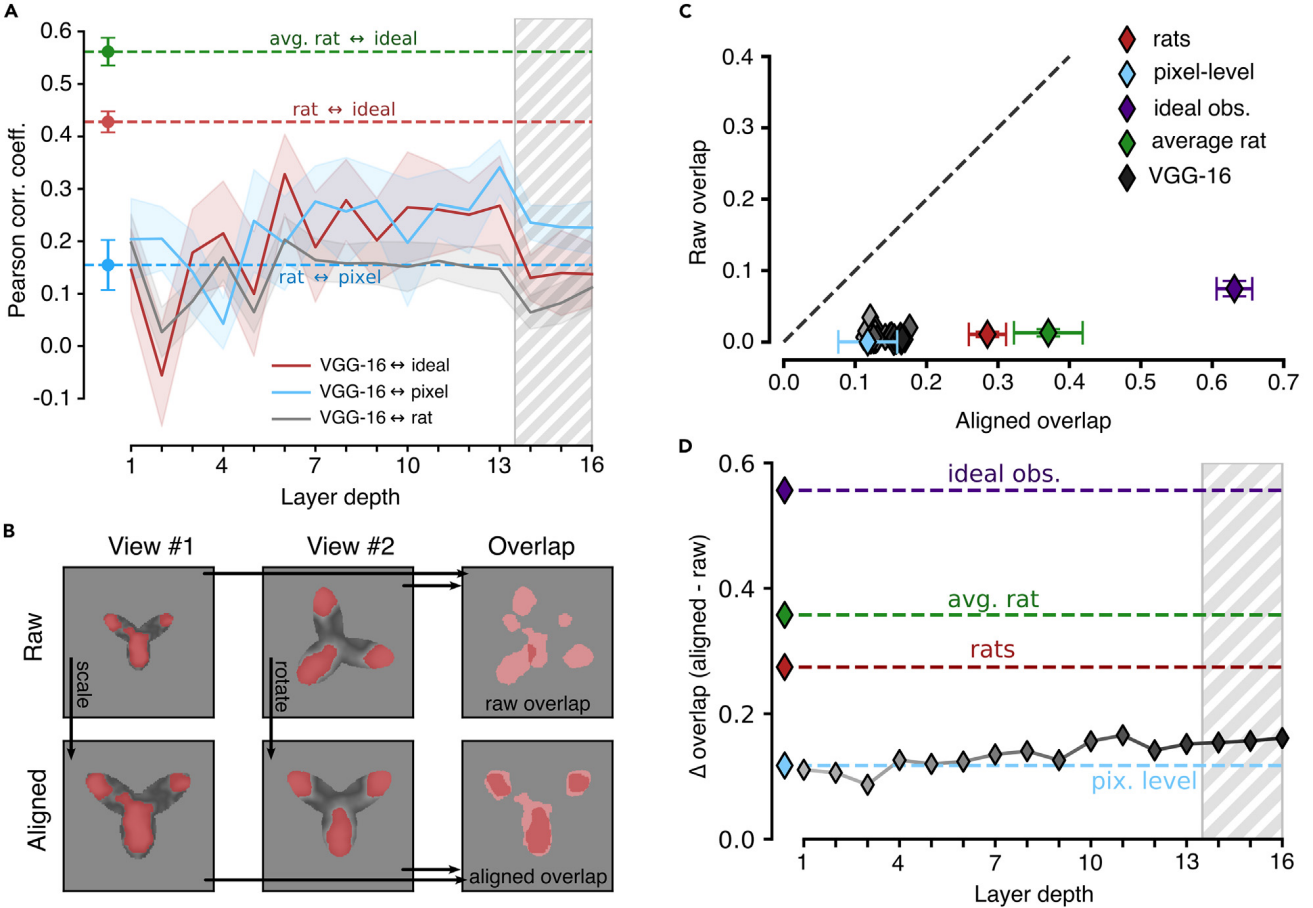
Superior view invariance of rat visual perceptual strategies (A) Pearson correlation coefficients between the saliency maps obtained for different pairs of “perceptual” systems: (1) rats vs. linear SVMs applied to VGG-16, layer-based representations (solid gray curve; the shaded area is the SEM across rats and object views); (2) rats vs. the ideal observer model (dashed red line; the error bar is the SEM across rats and object views); (3) average rat vs. the ideal observer model (dashed green line; the error bar is the SEM across object views); (4) rats vs. linear SVMs applied to the pixel-based representations (dashed cyan line; the error bar is the SEM across rats and object views); (5) SVMs applied to the VGG-16, layer-based vs. the pixel-based representations (solid cyan curve; the shaded area is the SEM across object views); and (6) SVMs applied to the VGG-16, layer-based representations vs. the ideal observer model (solid red curve; the shaded area is the SEM across object views). (B) Illustration of the procedure to compute the raw (top) and aligned (bottom) overlaps between the salient regions obtained for two different views of the tripod object (see main text). (C) Scatter plot reporting the aligned and raw overlaps of the salient regions obtained for the tripod object from the various “perceptual” systems under examination: (1) the rats (red); (2) the average rat (green); (3) the ideal observer model (purple); (4) the pixel-based representation (cyan); and (5) representations across VGG-16 layers, with the shades of gray (from light to dark) coding for the layer depth (from early to deep). Each point is the average over all the views of the tripods and all the rats (for the red point). Error bars are SEM (in the case of VGG-16 layers, SEMs are not reported for clarity). (D) Difference between the aligned and raw overlap for the various “perceptual” systems shown in (C).

These observations were quantified in Figure 6A, which reports the average Pearson correlation coefficient between the saliency maps obtained for the rats and those measured across VGG layers, with the average being computed across six rats and the eight views of the tripod object (gray curve). This correlation remained very low across the whole depth of the network, being close to the one measured between the saliency maps of the rats and the maps obtained for the pixel-based representation (cyan dashed line). By comparison, the correlation of rat saliency maps with those obtained for the ideal observer was substantially higher (about 0.4; red dashed line), and an even larger value was observed when the average rat saliency maps were considered, as originally done by Alemi-Neissi and colleagues^5^ (about 0.55; green dashed line). Not surprisingly, the correlation of VGG saliency maps with those of the ideal observer was much lower (red solid curve) and not different from that with the maps yielded by the pixel representation (cyan solid curve). This indicates that rats were better than VGG-16 at discovering those portions of the “tripod” object that are more diagnostic of its identity in the face of view changes and partial occlusion. The network appeared to slightly improve the optimality of its visual strategy across the convolutional layers (see the mild increase of the red solid curve) but never reached the same level of similarity with the strategy of the ideal observer as attained by the rats.

This discrepancy between rat and VGG perceptual strategies could be interpreted in different ways. If one considers CNNs as the benchmark model systems for advanced visual processing, failure of rat saliency maps to tightly align with those of VGG would imply that rats process visual objects using lower-level, less refined strategies, as compared to the network. This conclusion would be in agreement with recent reports suggesting that rats rely on coarser contrast features than do CNNs and primates.^63^ However, this interpretation would be at odds with the observation that rat perceptual strategies match those of the ideal observer model much better than VGG strategies do. Given that the ideal observer is, by construction, optimally view invariant, this would suggest a superior ability of rats at discovering and deploying transformation-tolerant perceptual templates.

To test this hypothesis, we measured the invariance across object views of the visual strategies inferred for the rats, the VGG representations, the pixel representation, and the ideal observer model. This property can be quantified by computing the extent to which the salient regions obtained for two different object views overlap. Critically, the overlap can be computed following two very different approaches, which allow distinguishing low-level, pixel-based image processing from invariant, feature-based recognition. As explained in Alemi-Neissi and colleagues^5^ and illustrated in Figure 6B, given the saliency maps obtained for two views of an object, one can simply compute their “raw” overlap over the image plane (rightmost image in the top row). Otherwise, one can first reverse the transformations that produced the two views, realigning them back to the default view of the object (in the figure, this process is indicated by the arrows connecting the scaled and in-plane rotated views of the tripod, on the top, to their realigned versions, on the bottom) and then compute the resulting “aligned” overlap of the corresponding saliency maps (rightmost image in the bottom row).

The two overlap metrics have a very different meaning. A large raw overlap indicates that the observer consistently relies on the same portion of the visual display to recognize the object, thus suggesting the existence of some screen-locked, transformation-preserved cues that remain diagnostic of object identity despite view changes. This means that the supposedly invariant task can be trivially solved using a screen-centered, pixel-based strategy, which is easy for the observer to discover and apply. By contrast, a large aligned overlap means that the observer consistently extracts from the objects the same visual features despite view changes, where “same” refers here not to absolute screen coordinates but to the position, size, and shape of the features relative to the structure of the object (e.g., the tips of the lobes of the tripod object). In other words, the aligned overlap measures the extent to which the visual strategy is object aware and view independent.

As shown by Alemi-Neissi and colleagues,^5^ in the case of the rats, the aligned overlap was substantially larger than the raw overlap. This was particularly evident for the tripod object (red diamond in Figure 6C), for which the raw overlap (on average, across view pairs) was virtually zero, while the aligned overlap was close to 0.3 (i.e., about 30% of the union of the saliency maps obtained for two views overlapped, on average). Based on this analysis, Alemi-Neissi and colleagues^5^ concluded that rat recognition could not be accounted for by a low-level, screen-centered strategy and relied instead on object-centered visual features that were fairly well preserved across transformations. Figure 6C further reinforces this conclusion by showing that, when the idiosyncratic strategies of the individual rats were combined in the group-average saliency maps (i.e., those shown in the second row of Figure 5), the aligned overlap became even larger (green diamond), approaching 0.4. This value was not too far from that obtained for the ideal observer model (about 0.6; purple diamond), which, not surprisingly, displayed the highest, “non-trivial” view invariance.

In our study, we replicated this analysis for the saliency maps obtained for the SVM classifiers trained on the pixel representation and on VGG layers’ activations. The aligned overlap obtained for the pixel-based maps (cyan diamond) was just one-third of the one observed for the individual rats and about one-fourth of that measured for the average rat. The aligned overlaps computed for the VGG maps (gray-shaded diamonds, with the depth of the network increasing from light to dark shades) were also very low in the initial layers and close to the overlap obtained for the pixel-based maps. Interestingly, despite a tendency to increase in deeper layers, the aligned overlaps remained substantially lower than the overlap measured for the rats, never surpassing 0.2.

These trends were further quantified by plotting the difference between the aligned and raw overlap as a function of the network depth (Figure 6D, gray curve). Although a modest increase could be observed, the network never reached the level measured for the individual rats (red dashed line) or for the average rat (green dashed line). The difference remained very close to the one observed for the pixel-based maps (cyan dashed line) and very distant from the one measured for the ideal observer (purple dashed line).

Overall, this analysis shows that, along the continuum that goes from the low-level, poorly invariant strategy of the pixel-based representation to the highly invariant perceptual templates of the ideal observer, the visual strategies afforded by VGG representations sit very close to the pixel representation. Rat perceptual templates substantially depart from such poorly invariant strategies and tend toward the maximally invariant templates attained by the ideal observer. The implications of this finding are examined in the discussion in the context of previous work comparing image processing in humans and CNNs.

### Rat perception is more invariant than VGG-16 representations to reduction of visual objects to their outlines

**Figure 7:**
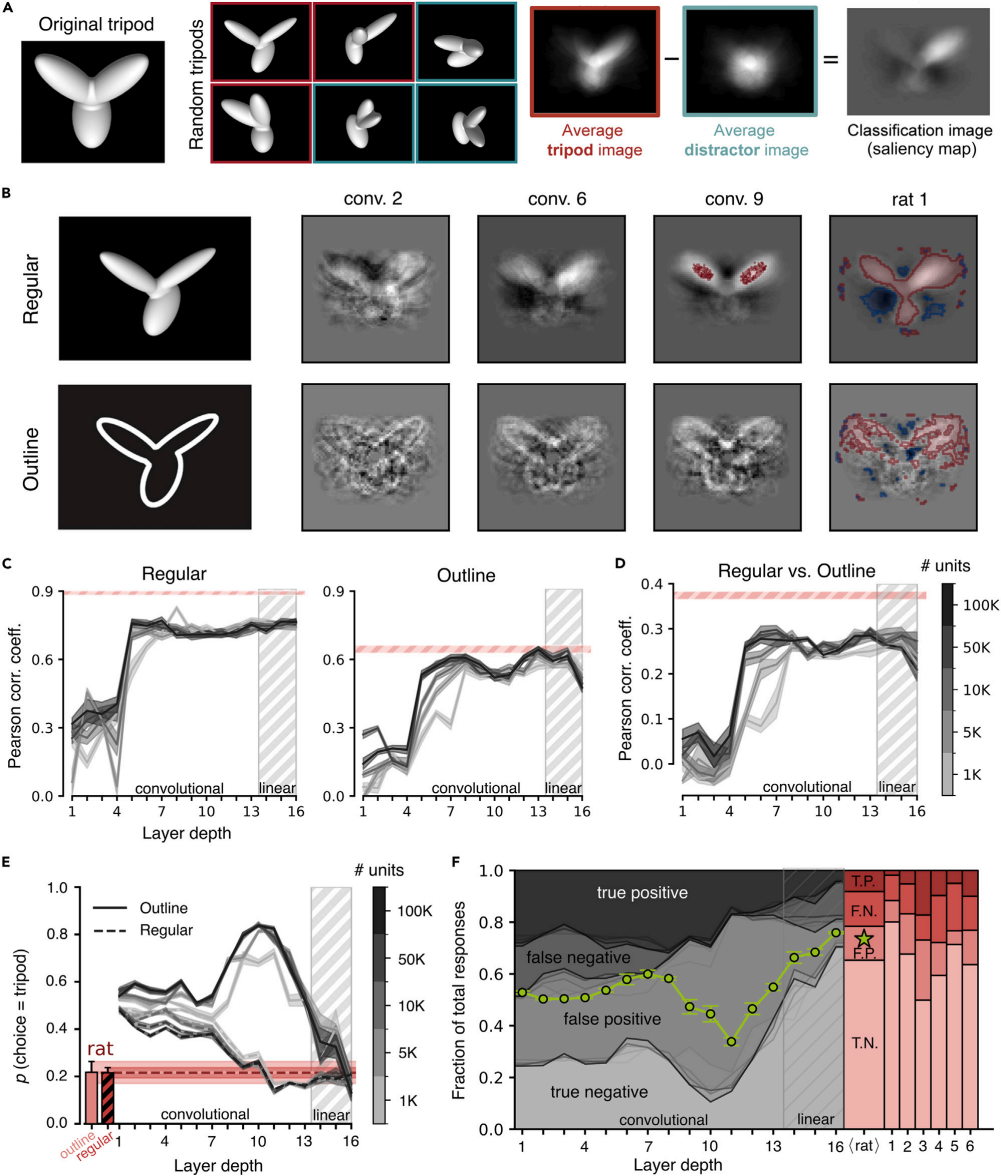
Superior generalization of rat perception to outline versions of visual objects (A) Stimulus set and experimental design originally used by Djurdjevic et al.^12^ Subjects were trained to recognize the tripod (left) from a set of distractor objects (not shown). They were then tested against random structural variations of the tripods. A saliency map (rightmost image) could thus be obtained by averaging the random tripods that were classified as the tripod (red frames) and subtracting the average of those that were classified as distractors (cyan frames). The same procedure was applied in our experiments with VGG-16. Adapted from Djurdjevic et al.^12^ (B) The saliency maps obtained for linear SVMs applied to the representations in three convolutional layers of VGG-16 are shown alongside the saliency maps of an example rat from Djurdjevic et al.^12^ The maps were obtained for two classes of stimuli: regular (i.e., full-body) objects and their outline versions (examples shown on the left). In the maps, light- and dark-gray regions refer to pixels that were, respectively, correlated and anti-correlated with reporting the “tripod” choice. The red and cyan contours mark regions for which such correlations and anti-correlations were statistically significant (p<0.01 on permutation test with 100 repetitions). (C) Pearson correlation coefficient between the saliency maps obtained for the rats and those computed for linear SVMs applied to VGG-16, layer-based representations using either regular (left) or outline (right) random tripods (solid gray curves; the shaded areas are SEMs across rats and classification runs). The shades of gray indicate the number of units that were sampled from each layer and fed to the SVMs (see color bar in E). The shaded red stripes mark the SEMs (centered on the averages) of the correlation coefficients between the saliency maps of the six rats tested by Djurdjevic et al.^12^ (D) Pearson correlation coefficient between the saliency maps obtained for linear SVMs applied to VGG-16, layer-based representations in the case of matching views of the regular and outline random tripods (the shaded areas are SEMs across classification runs). The shaded red stripe indicates the same analysis for the saliency maps obtained for the rats (SEM centered on the rat group average). (E) Fraction of random tripods that were classified as the tripod in the case of regular and outline objects (dashed and solid lines, respectively), by the rats (red bars/lines), and by linear SVMs applied to VGG-16 layer-based representations (gray curves). Shaded areas and shades of gray as in (D). (F) Visualization of the confusion matrix obtained when considering the classification of the regular random tripods performed by the rats (red bars) or by the SVMs applied to VGG-16 layer-based representations (gray curves) as the ground truth against which to compare the classification of the outline versions of the stimuli. The shade of gray of the curves indicates the number of units that were sampled from each layer and fed to the SVMs (see color bar in E). The green dots and the star show the classification accuracy achieved, respectively, by the SVM classifiers and by the rats (group average). Error bars are SEMs across classification runs.

An alternative classification image approach to infer the perceptual strategies underlying visual object recognition in rats has been developed by Djurdjevic and colleagues.^12^ In that study, the animals were initially trained to discriminate the tripod object from a set of distractor objects.^12^ Since the image of the tripod was rendered from a 3D model, its appearance could be parametrically altered by random variations of its structural parts, resulting in a new set of stimuli, referred to as “random tripods” (see examples in Figure 7A). Once a rat had learned to successfully discriminate the tripod from the distractors, it started to be presented also with the random tripods. The rats spontaneously classified these stimuli as belonging to either the tripod or the distractor category, based on how similar to the original tripod the objects were perceived. Importantly, the animals never received any feedback about the correctness of their choices. This allowed testing of their pure, spontaneous generalization on a new set of previously unseen images. In addition, the experimental design of the study by Djurdjevic and colleagues^12^ allowed probing the invariance of the generalization process, since the animals were also tested with scaled and outline versions of the random tripods (an example of the latter is shown in Figure 7B, left).

As in the study by Alemi-Neissi and colleagues,^5^ the ultimate goal of this experiment was to uncover the perceptual templates used by the rats to recognize the tripod object across transformations. To this aim, a saliency map was obtained from the pattern of responses of each animal to the random tripods by computing the difference between the average of the images (in the pixel space) that had been classified as the tripod and the average of those that had been classified as a distractor (see Figure 7A and methods). Each average was computed over a very large number of images (ranging between 900 and 3.500), thus yielding very sharp (i.e., high-contrast) saliency maps. Compared to the bubbles method used by Alemi-Neissi and colleagues,^5^ this approach had the advantage that it did not require masking the objects. This, in turn, allowed looking for salient an anti-salient features also outside the boundaries of the tripod. In addition, the resulting saliency maps were obtained from object conditions (the random tripods) that were better matched to the originally learned tripod in terms of both low-level (e.g., luminosity) and higher-order (overall geometry) properties.

Djurdjevic and colleagues^12^ tested in this experiment six rats, finding classification images with statistically significant salient regions typically encompassing two or three lobes of the tripod and anti-salient regions located at the lobes’ intersections (see the red and cyan regions in the map shown for an example rat in Figure 7B, last column). Small differences existed among the maps inferred for different animals, which were impactful enough to account for the different performances of the animals with the distractor objects. In addition, the saliency maps obtained for a rat from the regular, small, and outline versions of the random tripods were highly consistent, highlighting, once more, the invariance of rat recognition strategy.

In our study, we replicated the original experiment of Djurdjevic and colleagues^12^ in an artificial setting. We trained a linear SVM for each layer of VGG-16 to correctly discriminate the original tripod from the pool of distractor objects using the inner representation afforded by a subpopulation of units in each layer (as in our previous tests, subpopulations of different sizes were used). We then fed the network with the set of random tripods and recorded the classification labels assigned by the SVMs to these stimuli in a pure generalization setting that matched the test applied to the rats. Following a procedure analogous to the one presented in Djurdjevic and colleagues,^12^ we computed, for each layer of the CNN, a saliency map that revealed which visual features were critical for successful discrimination of the tripod from the distractor objects.

Example saliency maps for the regular (i.e., full body) and outline conditions are reported in Figure 7B for three convolutional layers of VGG-16 alongside the saliency maps of an example rat. From visual inspection, the saliency maps obtained from VGG representations seemed to progressively converge to the maps measured for the rats. To quantify this intuition, we computed the average Pearson correlation coefficient between VGG and rats’ saliency maps as a function of the depth of the network (Figure 7C, gray lines). We also computed the average Pearson correlation coefficient among the maps of the six rats to serve as a consistency benchmark (the red shaded area in the figure indicates the region encompassed by ±SD over such an average, rat-wise correlation).

For the maps extracted from the responses to the regular random tripods (Figure 7C, left plot), the correlation started low and then increased sharply from layer 4 to layer 5, reaching a plateau that was remarkably stable over the rest of the network—a finding that was robust across the different population scales we explored. Differently from what observed in the comparison with the study of Alemi-Neissi and colleagues^5^ (see Figure 6A), this asymptotic correlation was high (around 0.7), thus showing that middle to late VGG layers yielded discriminatory visual features that were quite consistent with those of the rats. At the same time, such consistency did not reach the one among rat maps, whose correlation was close to 0.9. This indicates that, even in deeper layers, VGG saliency maps did not fully capture the perceptual strategies deployed by the rats. The correlation between VGG and rats’ saliency maps followed a similar trend also in the case of the outline stimuli (Figure 7C, right). In this case, however, the maximal correlation matched the one measured among the rats. In addition, following an initial plateau in the middle of the network, the correlation slightly decreased to further grow in the final convolutional layers.

As already mentioned, Djurdjevic and colleagues^12^ found that the saliency maps obtained from the various transformed versions of the random tripods were quite consistent, even in the case of radical transformations such as the change from regular to outline stimuli. Such consistency is quantified in Figure 7D, which reports the within-rat correlation between the saliency maps yielded by regular and outline random tripods averaged across the six animals (red shaded stripe). The magnitude of the correlation approached 0.4, which is no small value considering how different the stimuli yielding the two kinds of saliency maps were and, as a result, how scattered the outline-based saliency maps were, as compared to the regular-based maps (contrast the two images in the last column of Figure 7B). The same correlation metric was computed between the maps obtained from regular and outline stimuli for each layer of VGG-16 (gray curves). The correlation sharply increased between layers 5 and 7 to reach a relatively stable plateau but failed to match the value measured for the rats, even in deep layers. This confirmed the conclusion of the previous section, i.e., that rats employ perceptual strategies that are more invariant to challenging image transformations than those afforded by a fully trained CNN.

As a final step in our analysis, we took inspiration from one of the results presented in the previous sections, namely the poor generalization of VGG representations to the partially occluded object conditions (star symbols in Figure 4B). This finding suggests that, despite its proficiency with challenging image sets such as ImageNet, VGG-16 is quite sensitive to severe image manipulations—possibly those that drastically alter the surface area of the objects, strongly reducing their luminosity, thus yielding out-of-distribution samples. The stimulus set of Djurdjevic and colleagues^12^ offered the possibility to further investigate this phenomenon, given that transforming the random tripods into their outlines produced image changes as severe as the application of the bubble masks. Quite impressively, for the rats these changes did not alter at all the average likelihood of classifying a random tripod as being the tripod, as opposed to belong to the distractors’ category. As shown by Djurdjevic and colleagues,^12^ the probability that a rat classified a random tripod as being the tripod was equally low (about 0.2) and virtually identical for both the regular and the outline stimuli (compare the red to the striped bar in Figure 7E). This suggests that rats developed perceptual templates that were sharply tuned to the shape of the tripod and that such templates were fully invariant to the transformation that changed the regular, full-body stimuli into their outline counterparts.

When we applied this analysis to the choices of the SVM classifiers trained on the tripod vs. distractor task based on VGG representations, we found several important differences with rat behavior. Up to the middle of the network, the probability to classify a random tripod as being the tripod (gray lines in Figure 7E) was similar for the regular and outline stimuli, but much higher (around 0.5) than the one measured for the rats. This indicates that reading the activations of VGG units in early to middle layers did not allow the linear classifiers to find regions within the representational spaces that were specific enough for the shape of the tripod to not include most of its random, structural variations. Interestingly, starting from layer 8, the proportions of tripod choices for the outline and regular stimuli diverged, reaching, in layers 10 and 11, extremely large (0.8) and low (⟨0.2) values, respectively. This indicates a complete lack of invariance in late convolutional layers to the outline transformation. Only in the very final convolutional and fully connected layers the probability of tripod responses to the outline stimuli dropped while that to the regular stimuli slightly increased, with both probabilities eventually converging on those measured for the rats.

To further investigate these trends, we also measured how consistent the classification of every random tripod was when presented in its regular and outline version. While no correct label existed for the classification of the random tripods, one can quantify the consistency between the responses to the two variants of these stimuli (regular and outline) by considering the decisions for a given variant as the “ground truth” and then checking whether the choices for the other variant are consistent with these “true” labels. In our analysis, we used the classification of the regular random tripods to define their “correct” labels and tested whether the classification of the same random tripods, but in their outline version, was in agreement with these labels. This yielded the confusion matrices shown in Figure 7F for VGG layers (gray shaded areas) and for the rats (red shaded bars). For the latter, despite some variability across animals, the classification accuracy (i.e., the consistency between responses to regular and outline random tripods) was high, with the sum of true negatives (i.e., random tripods classified as distractors in both their regular and outline variants) and true positives (i.e., random tripods classified as the tripod in both their regular and outline variants) being close to 70% on average across rats (green star). In the case of the network, classification accuracy was substantially lower until the very final layers (green line), mainly because of the large rate of false positives and the concomitantly low rate of true negatives. Performance was especially low in layers 9–11, where it went below chance, reaching a minimum in layer 11. This indicates that representations in those layers supported opposite choices when the random tripods were shown as outlines rather than as full-body objects (in agreement with the strong divergence in the proportion of tripod choices for the two kinds of stimuli illustrated in Figure 7E). Starting from layer 12, performance increased abruptly, until the deepest, fully connected layer eventually displayed a confusion matrix that closely matched that of the average rat.

Mechanistically, it is not easy to understand the causes of this non-monotonic trend. One possible explanation comes from previous studies^56^^,^^64^^,^^65^ showing that, in deep CNNs, category-specific information does not develop gradually along the layers’ progression but emerges quite abruptly toward the end of the convolutional architecture, with a sharp rise starting at layers 11–12 in the case of VGG-16. This has been linked to the need of pruning information about features that are irrelevant for the classification and reformatting the representations in such a way as to make the category-relevant information more explicit. What Figures 7E and 7F suggest is that this process is not equally difficult for full-body and outline objects. With the former, the network already achieves a stable classification in the middle of the network (i.e., the fraction of tripod choices remains around 20%, close to that of the rats, from layer 8 onward). By contrast, with the outline stimuli, there is a need to prune more irrelevant information before useful, category-specific information becomes explicit, and this only starts happening after layer 11, when choices on the outline random tripods take a sharp turn that will eventually bring them to alignment with choices on the regular, full-body stimuli. Overall, this further reinforces the conclusion that VGG-16 achieves the same level of invariance of rat object vision only in its final layers under the most challenging, out-of-distribution image manipulations.

### Comparison with an untrained VGG-16 and a size-matched multi-layer perceptron

All the comparisons presented above were carried out using VGG-16 pre-trained on ImageNet, a very large and diverse set of natural images. The representations resulting from such training are obviously specialized to effectively classify these images, although only the very final convolutional and fully connected layers encode category-specific information, while previous convolutional layers appear to encode general-purpose visual features.^26^^,^^54^^,^^56^^,^^65^ Thus, one may ask how critical it is for the network to be trained with such a rich image set in terms of its ability to account for rat visual perception. The answer to this question is not obvious, because CNNs have built-in architectural priors (convolutional filters, pooling filters, and feedforward hierarchy) that make them powerful processing machines for image detection, classification, and denoising even in the absence of training.^66^^,^^67^^,^^68^^,^^69^

**Figure 8:**
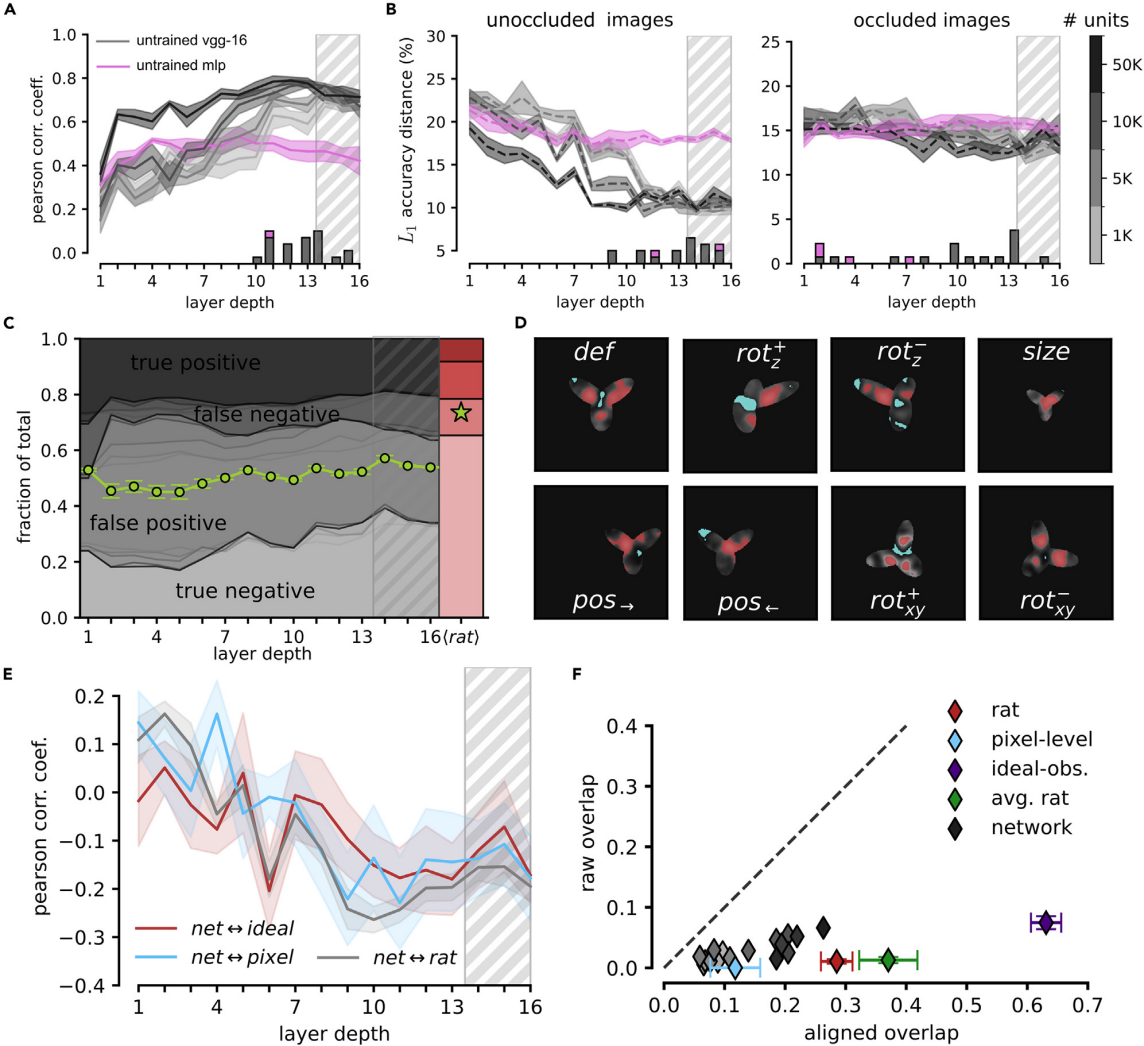
Comparison with an untrained VGG-16 and an untrained multi-layer perceptron (A) Same analysis as in Figure 3F, but for a randomly initialized VGG-16 (shades of gray; see color bar in B) and a randomly initialized multi-layer perceptron (MLP; pink). (B) Same analyses as in Figures 4C and 4D, but for a randomly initialized VGG-16 (shades of gray) and a randomly initialized MLP (pink). (C) Same analysis as in Figure 7F, but for a randomly initialized VGG-16. (D) Saliency maps obtained for the SVM classifiers trained on the representations provided by the last convolutional layer (i.e., layer 13) of a randomly initialized VGG-16. Same color code as in Figure 5. (E and F) Same analyses as in Figures 6A and 6C, but for the saliency maps yielded by consecutive layers of a randomly initialized VGG-16. See Figure S1 for further details.

Our tests with an untrained VGG-16 (i.e., with the network having randomly assigned weights) are reported in Figure 8. When probed with objects undergoing shifts, scaling, and rotations, representations along consecutive layers of the network yielded patterns of discrimination accuracies that became progressively closer to those observed for the rats (Figures 8A and 8B, left; gray curves). For the dataset of Zoccolan and colleagues,^4^ the correlation with rat performance reached a peak as high as the one obtained for the trained VGG-16 (i.e., 0.8), but in the deepest convolutional layers rather the in the middle of the network (compare Figures 8A and 3F; compare also Figures S1A and 3D). Similarly, for the dataset of Alemi-Neissi and colleagues,^5^ the minimal $L_{1}$ distance was as low as for the trained network, but it was reached only in the final convolutional layers rather than in layer 8 (compare Figures 8B [left] and 4C; compare also Figures S1B and 4B). By contrast, the untrained VGG performed very poorly with the occluded images, never reaching the same accuracy attained by the rats (Figures 8B [right] and S1B [stars]; compare to Figures 4D and 4B, stars). It performed similarly poorly when we compared how the full-body and outline random tripods used by Djurdjevic and colleagues^12^ were classified by the network, with the consistency of the classification remaining close to chance across the whole depth of processing (Figure 8C, green line; compare to Figure 7F).

Overall, this shows that the hierarchical increase in the complexity of the convolutional filters, although randomly assembled, and the progressively larger invariance endowed by the pooling filters are enough for the network to achieve rat-like performance patterns, but only with object transformations that are not too challenging (i.e., no occlusion and no reduction to outline) and only at the very end of the processing hierarchy. This result is far from surprising if one considers that, before the advent of deep learning, multi-layer feedforward networks such as HMAX, alternating convolutional kernels and max pooling, were the best models of the ventral stream^70^^,^^71^^,^^72^^,^^73^^,^^74^^,^^75^ and powerful machine vision systems,^73^ despite being untrained. In other words, an untrained CNN cannot be taken as a null model against which to compare rat vision. We reasoned that an appropriate negative control would instead be an untrained multi-layer perceptron (i.e., a deep, fully connected feedforward net) matched to VGG-16 in terms of the number of free parameters and layers (see methods). This network proved unable to match rat accuracy and its modulation across transformations, showing no hierarchical increase across layers (Figures 8A, 8B, S1A, and S1C; pink curves). This indicates that rat object vision cannot trivially be modeled by implementing a cascade of random projections of the input images in high-dimensional representational spaces. If a deep feedforward network is untrained, it can account for some aspects of rat vision only if it implements a hierarchical buildup of selectivity and invariance via built-in architectural priors such as convolutional and pooling filters.

To further investigate the extent to which these computations alone can account for rat visual perception, we also looked at the visual discrimination strategy of the untrained VGG-16. Differently from the case of the trained network, the saliency maps obtained in the final convolutional layer were much more similar across transformations (Figure 8D; compare to Figure 5). At the same time, the salient regions were located at the base rather than at the tip of the lobes, as found for the rats. Both trends emerged gradually across the depth of the untrained net. The saliency maps become increasingly anti-correlated with those of the rats as well as with those of the ideal observer and of the pixel-based representation (Figure 8E; compare to Figure 6A). They also become more view invariant, reaching an aligned overlap value close to that of the rats, although with a larger raw overlap (Figure 8F). This suggests that training, while yielding larger discrimination accuracies, pushes the network to rely on more view-specific strategies (see discussion).

### Comparison with VGG-16 trained with blurred images

In our experiments, we accounted for rat low visual acuity via an image-augmentation pipeline that included a Gaussian-blurring step before images were fed to VGG-16 (see Figures 1 and 2). Such manipulation inevitably shifted the power spectrum of the test images away from the distribution to which the network was exposed during training, and one may wonder whether this shift affected the way VGG-16 represented the stimuli used in the rat studies. In other words, one may ask whether training the network with blurred images would make it more suited to represent the low-pass-filtered images used in our tests and, as such, a better model of rat vision.

To address this point, we leveraged recent work showing that training CNNs with blurred images improves their robustness to noise and their ability to model human vision.^76^ Specifically, we used the VGG-16 network trained by Jang and Tong^76^ in the strong-blur regime, in which images sampled from the 1,000 ImageNet categories were pre-processed with a Gaussian blur filter whose *σ* was uniformly sampled in σ∼{0,1,2,4,8} pixels. In our tests, a full-screen image fed to the network had a size of 224×224 pixels and would span $∼96^{\circ }$ of visual angle in the behavioral rigs used in the rat experiments (Figure 2E). The set of blur intensities applied by Jang and Tong^76^ would thus correspond to a range of cutoff frequencies $f_{\text{cut}}\in \left\{f_{\min},f_{\max}\right\}$, bounded from below by the heavy-blur condition at $f_{\min}∼0.06$ cycles/deg and from the top by the light-blur condition at $f_{\max}∼0.5$ cycles/deg. This range is nicely centered on the region of highest sensitivity of the rat contrast sensitivity function (see Figure 2A, black curve). It includes the spatial frequency of 0.1 cycles/deg, which corresponds to rat peak sensitivity and guarantees that the spatial frequency content of most training images was attenuated below 50% amplitude for frequencies higher than 1 cycle/deg (corresponding to rat highest spatial resolution). As such, the strong-blur regime implemented by Jang and Tong^76^ is a good approximation of the blurred visual world experienced by rats.

**Figure 9:**
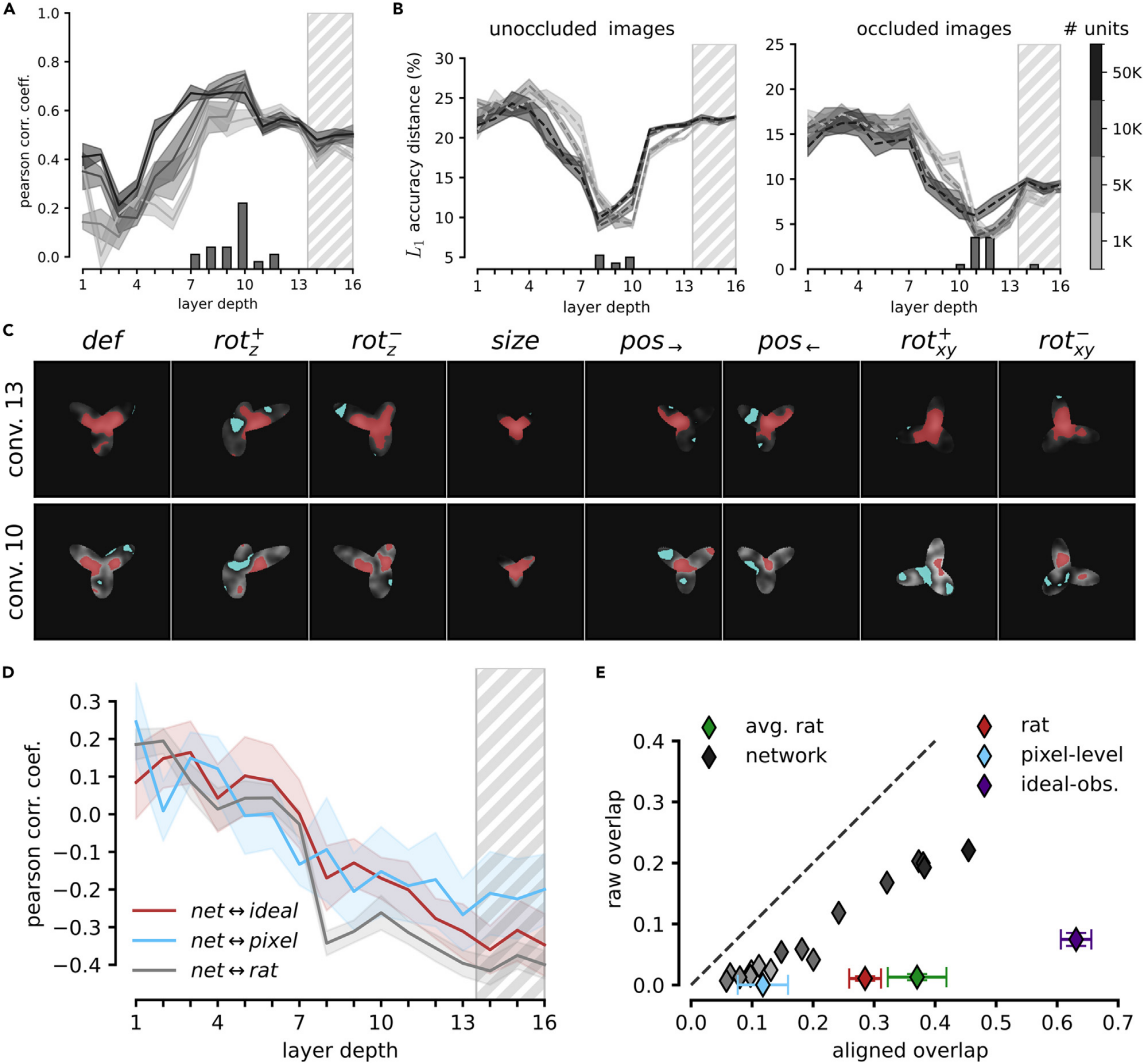
Comparison with VGG-16 trained with blurred images (A) Same analysis as in Figure 3F, but for a VGG-16 trained with the strong blur augmentation implemented by Jang and Tong.^76^ (B) Same analyses as in Figures 4C and 4D, but for a VGG-16 trained with strong blur augmentation. (C) Saliency maps obtained for the SVM classifiers trained on the representations provided by two-example convolutional layer of a VGG-16 trained with strong blur augmentation. Same color code as in Figure 5. (D and E) Same analyses as in Figures 6A and 6C, but for the saliency maps yielded by consecutive layers of a VGG-16 trained with strong blur augmentation.

Our tests with VGG-16 trained in this strong-blur regime are reported in Figure 9. When probed with objects undergoing shifts, scaling, and rotations, the layers where the patterns of discrimination accuracies best matched those of the rats did not differ substantially with those previously reported for VGG-16 trained with the regular ImageNet (compare Figures 9A and 9B [left] to Figures 3F and 4C). However, the match took place in slightly deeper layers, indicating that “living” in a visual world as blurred as that of the rat requires a slightly larger depth of processing to match rat perception. Conversely, and quite interestingly, the best match with rat discrimination of the occluded images occurred earlier, between convolutional layers 11 and 12 (Figure 9B, right), as opposed to the final, fully connected layers (compare to Figure 4D). On the one hand, this finding is consistent with the results of Jang and Tong^76^—partial occlusion being a form of noise, their network displays better robustness to such manipulation than a regular VGG-16. On the other hand, this suggests that low visual acuity can be one of the factors contributing to the strong tolerance rats display to partial occlusion (see discussion).

Also for the VGG-16 trained in the strong-blur regime, we looked at the discrimination strategies afforded by object representations across consecutive layers (see examples in Figure 9C). Differently from what was observed for rats (compare to Figure 5), the salient regions clustered at the base rather than at the tip of the lobes. These saliency maps were reminiscent of those observed with the untrained VGG-16 (compare to Figure 8D), but now, in the last convolutional layer, the salient patches merged at the intersection of the lobes, forming a single region located at the center of the object. Not surprisingly, these saliency maps were strongly anti-correlated with those of the rats, with the magnitude of the negative correlation steadily increasing along the depth of the network (Figure 9D; compare to Figure 6A). At the same time, we also observed a progressive and substantial increase of both the raw and aligned overlaps (Figure 9E). The latter, in the last fully connected layer, even surpassed that of the average rat (green diamond). While this could be taken as an indication of a discrimination strategy as view invariant as that of the rats, the fact that also the raw overlap reached very large values (in the case of the rats it was close to zero) implies that a low-level strategy, based on the detection of a transformation-invariant, screen-centered image patch, cannot be ruled out. In other words, while training with blurred images improved VGG-16 robustness to occlusion, making it more “rat-like” in this respect, when it came to the complexity and invariance of the perceptual strategy, the rats still stood out. None of the variants of VGG-16 probed in our study displayed rat ability to rely on multiple distinct features with a minimal screen-centered but a substantial object-centered overlap (see discussion).

### Impact of not applying the augmentation pipeline to the images fed to VGG-16

As previously mentioned, Vinken and Op de Beeck^28^ already assessed how well the representations along VGG-16 (pre-trained on ImageNet) support the discrimination of the objects used by Alemi-Neissi and colleagues^5^ in their rat experiments. Differently from what was found in our tests (Figure 3), they reported that the very first layer of VGG-16 was sufficient to achieve (on average, across all the off-cross views shown in Figure 3A) a discrimination accuracy that was beyond the average performance attained by the rats on those conditions (i.e., about 70% correct; see the red line in Figure 3D). Our hypothesis is that the discrepancy between our results and those of Vinken and Op de Beeck is due to the lack, in their tests, of an image-augmentation pipeline such as the one we implemented (see Figure 2). This prevented them from taking into account the actual amount of image variation, blur, and noise experienced by the animals, resulting in a task that was not consistent with that faced by the rats.

**Figure 10:**
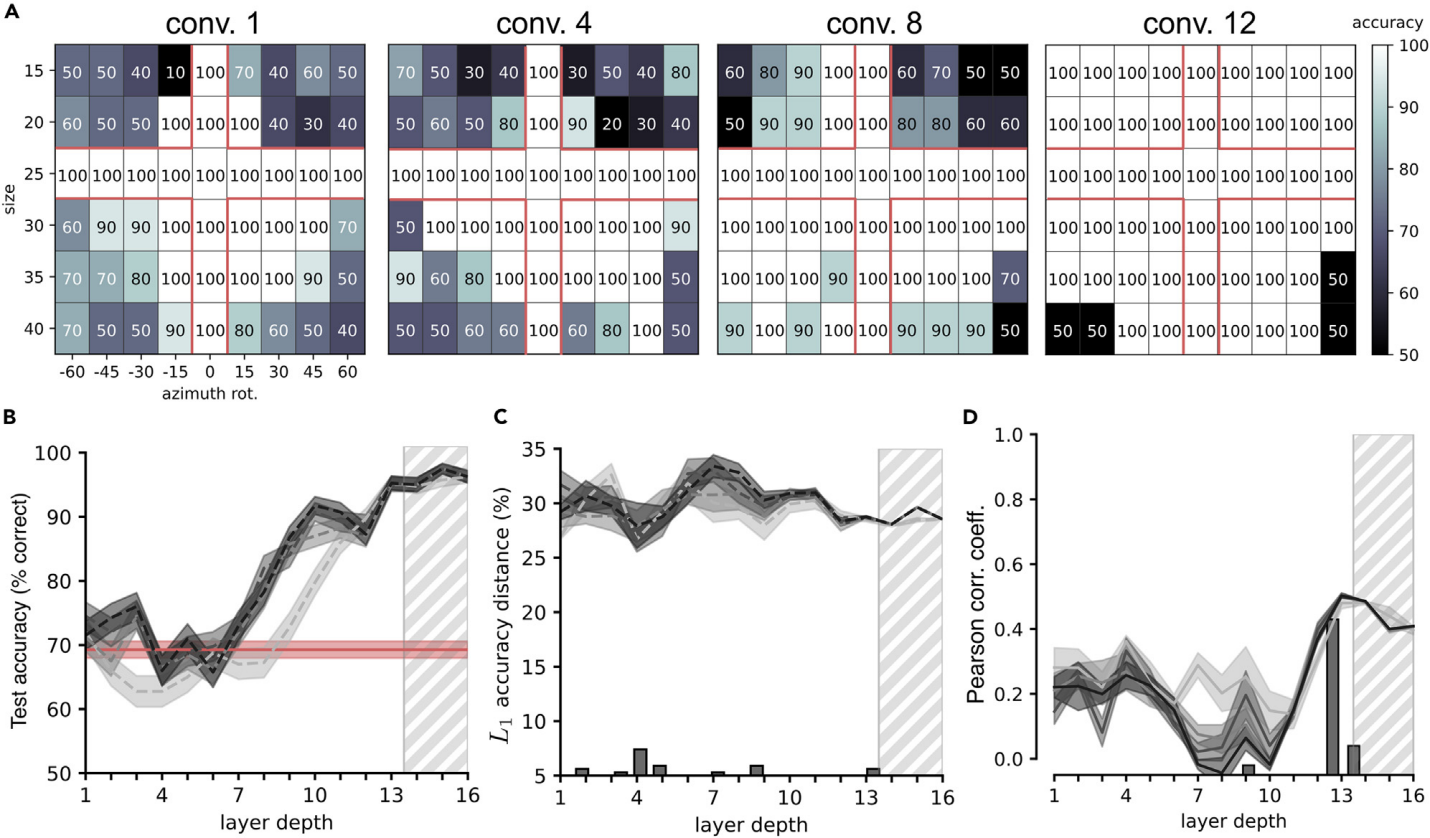
Simulating the amount of image variation, noise, and blur experienced by rats is critical to properly account for their patterns of discrimination accuracies (A) Same as in Figure 3C, but for a VGG-16 that was fed with images that were not subjected to the pre-processing augmentation pipeline described in Figure 2. (B–D) Same as in Figures 3D–3F, but for a VGG-16 that was fed with images that were not subjected to our pre-processing augmentation pipeline.

To verify this hypothesis, we repeated the analysis of Figure 3 without applying the pre-processing augmentation pipeline to the images that were fed to VGG-16. The resulting patterns of discrimination accuracies over the matrix of object transformations that we measured along the network are shown in Figure 10A for a few checkpoint layers. These patterns are substantially different from those reported, for the same layers, in Figure 3C (i.e., when the pre-processing pipeline was applied). While performance (over the whole matrix) started close to chance in Figure 3C, increasing gradually across layers and converging (in middle layers) to a modulation profile quite similar to that of the rats (shown in Figure 3B), in Figure 10A the discrimination accuracy was already maximal in the first convolutional layer for the views that were closer to the training conditions (i.e., to the on-cross cells of the matrix). At the same time, accuracy on conditions at the top corners of the matrix (i.e., very far from the training views) was very low (close to chance). This step-like modulation pattern, which remained almost unchanged until the middle of the network (layer 8), was substantially different from that observed for the rats, whose performance never saturated to 100% correct and decreased smoothly as a function of the distance from the training conditions but remained well above chance also in the top corners of the matrix (Figure 3B).

These qualitative observations are quantified in Figures 10B and 10C. Consistently with the results of Vinken and Op de Beeck,^28^ and differently from what was previously shown in Figure 3D, the average discrimination accuracy over the off-cross test views was already above the performance of the rats in the first convolutional layer (Figure 10B). The accuracy remained at that level until the middle of the network and then increased steeply toward perfect discrimination starting from layer 8. More interestingly, the distance between the accuracy matrix obtained for the rats and the accuracy matrices observed across consecutive layers of VGG-16 remained very large along the whole the depth of the network (Figure 10C; compare to Figure 3E) and, conversely, their correlation remained very low (Figure 10D; compare to Figure 3F). This corroborates the qualitative observation that no layer of the network yielded a pattern of discrimination accuracies matching that of the rats.

Overall, these tests help to explain the discrepancy with the results of Vinken and Op de Beeck^28^ and strongly support the need for matching as closely as possible the actual amount of image variation, noise, and blur experienced by the rats at the front end of their visual system before trying to model their visual behavior with a CNN. In our tests, only when such an image pre-processing pipeline was applied could a very good match between the accuracy patterns obtained for the rats and some layers of VGG-16 be found (see Figures 3E and 3F).

## Discussion

### Modeling rat visual perception using a deep convolutional network

The goal of our study was to evaluate the processing power of rat object vision by carrying out a comparison with a popular CNN, VGG-16, pre-trained on ImageNet. Our approach was similar to the one adopted in previous CNN-based assessments of the proficiency of humans and rats to recognize visual objects.^27^^,^^28^ We estimated how difficult it was for a rat to succeed in a given object-recognition task by interrogating object representations across VGG layers to test how well they supported the task ("accuracy measurement" pipeline in Figure 1). The depth of the layer yielding the best match with the pattern of rat discrimination accuracies was taken as a measure of how advanced the processing carried out by the animals was to succeed in the task. However, differently from Vinken and Op de Beeck,^28^ our approach included: (1) a pre-processing/augmentation stage that closely reproduced the blurring, noise level, and image variability experienced by the animals in the tasks (Figure 2); (2) a focus on comparing how similarly recognition accuracy was modulated across object views in rats and CNN layers rather than a comparison of absolute discrimination performances; and (3) a comparison between the “perceptual” strategies used by rats and CNNs to solve the same object-recognition task ("strategy inference" pipeline in Figure 1).

Before discussing our findings, it is worth mentioning that our approach rests on a well-established and very successful rationale whereby deep CNNs, pre-trained with large sets of natural images (most often ImageNet), are used to model either visual perception or the tuning of visual neurons.^23^^,^^24^^,^^77^ At the root of this approach there is the observation that, as a result of the training, deep CNNs learn powerful, general-purpose representations of the visual features/patterns/shapes/textures found in the natural world. These representations are so general, rich, and diverse that they are able to account for perceptual choices and neuronal tuning, even if the latter are probed with stimuli that are not sampled from the pool of training images (i.e., are not sampled from ImageNet). In other words, modeling perceptual and neurophysiological data exploits so-called transfer learning, a very popular approach in the field of machine learning whereby a pre-trained network with, e.g., ImageNet is finely tuned to solve another task (e.g., one based on a different image set) by simply retraining the last, fully connected readout layer.^54^ The difference is that, in brain modeling studies, such retraining is applied to the final readout layer only when the goal is to carry out a comparison with the image-classification ability of the complete network.^19^^,^^20^ In many cases, however, the goal is to find which depth of processing (i.e., which layer) yields the best fit with the psychophysical or neuronal data.^21^^,^^25^^,^^26^^,^^27^^,^^28^^,^^78^ Hence, the readout stage to be finely tuned via transfer learning can be mounted on top of any layer of the network, since the goal is to carry out a comparison with processing up to that checkpoint depth. Also, the readout stage does not need to be a fully connected perceptron-like layer. Most often, the specific categorization task is learned by applying to the representation in the checkpoint layer a multivariate linear regression or a linear decoder (typically a linear SVM, as done in our study). The important aspect is for the readout stage to be linear. This choice not only makes it consistent with a possible neuronal implementation but also guarantees that the readout process is simple enough—i.e., just a way to assess what the representation in the checkpoint layer is capable of and not a means to add further (advanced) processing on top of the one already carried out by the network up to that layer.

These considerations also help the understanding of how the results of modeling brain processes with CNNs have to be interpreted. Finding that a CNN (or a portion of a CNN) well accounts for a perceptual performance or neuronal tuning does not imply that the brain under study and the CNN learned visual representations in the same way. In fact, while CNNs are typically trained in a supervised way using millions of labeled images and by minimizing a global cost function,^79^ learning in visual cortex is thought to rely mostly on local, unsupervised plasticity rules that mold visual representations to the statistical structure of the visual environment.^80^ Therefore, what a good match between CNN and brain representations (or performances) indicates is that the processing carried out by the visual system is consistent with a hierarchical feedforward architecture, where some key computations (convolution and pooling) are iterated. Learning plays the important role of finely tuning these computations (e.g., by determining the structure of the convolutional kernels) to better fulfill the needs of the perceptual system. In this sense, finding a good match between processing in the brain and in a CNN strongly suggests that, because both systems had to solve similar tasks (e.g., object recognition), they developed similar shape representations, despite major differences in the learning algorithms and the image sets to which they were exposed.

Given these premises, what our study shows is that the accuracy patterns reported in several previous studies of rat object vision are consistent with the processing carried out by a hierarchical feedforward network, where feature selectivity and spatial invariance are built using convolutional and pooling kernels. We found that the depth of processing that best matched rat perception could vary depending on the kind of manipulations applied to the test images and the sort of training the CNN model received, but it never trivially spanned just a few layers. All the variants of VGG-16 we tested (trained or untrained; trained with or without blurring) required at least half the computational depth to provide a good match with rat discrimination accuracy. This means that succeeding in the discrimination was not a trivial task for the animals, a conclusion reinforced by the fact that the sophistication and invariance of rat recognition strategy were unmatched by the network, even in the deepest layers.

### Summary and implications of our findings

Our experiments yielded three main results. First, we found that the patterns of rat classification accuracy in tasks where objects underwent changes in position, size, and in-depth and in-plane rotation are best accounted for by representations in VGG-16 mid-level layers (Figures 3E, 3F, 4B, and 4C). This conclusion is in disagreement with the study of Vinken and Op de Beeck,^28^ who found that the very first layer of VGG-16 and AlexNet (an older, shallower CNN) was sufficient to achieve nearly perfect discrimination accuracy (i.e., well beyond that attained by the rats) in the object-recognition task reported by Zoccolan and colleagues.^4^ We verified that such discrepancy is due to the underestimation of the actual image-level variability, noise, and blurring experienced by the rats in the analysis of Vinken and Op de Beeck^28^ (Figure 10). On the other hand, Vinken and Op de Beeck^28^ also tested the extent to which the representations in CNN layers were directly able to account for the pattern of rat behavioral accuracies over the tested object views, as reported by Zoccolan and colleagues^4^ (i.e., the matrix of performances shown in Figure 3B). The rationale of this analysis is more consistent with that of our approach and, not surprisingly, the tests of Vinken and colleagues showed that mid- to high-level convolutional layers better accounted for the modulation of rat performance across object conditions than early layers.

The second major finding of our study is that the entire depth of VGG-16, up to the final, fully connected layers, was required to achieve the best match with rat ability to generalize to more radical image manipulations of visual objects: heavy occlusion and reduction of an object to its outline (Figures 4D, 4E, 7E, and 7F). In addition, generalization to occluded images was not achievable without including them in the training diet of the SVMs so as to avoid degenerate performances on these stimuli. Moreover, while an untrained VGG-16 still succeeded at matching rat perception (albeit in the deepest convolutional layers) when the objects underwent changes in position, size, and rotation (Figures 8A and 8B, left), discrimination of the occluded and outline objects remained close to chance across the whole depth of the network in the absence of training (Figures 8B [right] and 8C; see also Figure S1B). By comparison, the robustness of rat perception to these transformations (Figures 4B, 7E, and 7F) indicates that, when visual patterns are incomplete and undergo severe variations of luminosity, the relatively shallow rat visual cortical hierarchy^7^^,^^15^^,^^81^ displays all its generalization power, well beyond what is revealed when the animals are tested with spatial transformations and rotations or even with naturalistic movies,^28^ and well beyond what is achieved by VGG-16 early- to mid-level convolutional layers.

It could be argued that this ability lies in the pruning of low-level visual properties (such as luminance and contrast) that is carried out by the rat object-processing pathway.^7^ However, we recently reported evidence for a similarly strong pruning taking place along VGG-16 early layers.^56^ Thus, it is more likely that rat robustness to occlusion resides on processing mechanisms that are not purely feedforward, in agreement with the conclusions of previous studies suggesting a pivotal role of recurrent computations in supporting pattern completion.^82^^,^^83^^,^^84^^,^^85^^,^^86^ Interestingly, some of these studies showed that CNNs are not as tolerant as the human visual system to partial occlusion, but their robustness to this manipulation was substantially increased by the addition of recurrent connectivity.^83^^,^^86^ Since a recurrent neural network can be unfolded by adding extra layers for each recurrent step to create a computationally equivalent feedforward network,^87^ this can explain why the full depth of VGG-16 is necessary to match what the rat visual cortex achieves with much fewer (but highly recurrent) processing stages—i.e., 6–7 visual areas from retina to the highest cortical areas of the object-processing pathway.^7^^,^^15^^,^^81^ These considerations are also in agreement with neurophysiological studies of the invariance of high-level representations to image transformations in primates. Several authors found that different kinds of transformations require a different depth of processing along the visual hierarchy and/or a different latency to fully develop. For instance, Murty and Arun^88^ reported that tolerance of IT neurons to in-plane and in-depth rotations is weaker than to size and position changes (a trend they also found in a CNN) and takes longer to emerge. A similar finding had been previously reported along the human ventral stream, where size- and position-invariant object information was found to develop in stages, with latency growing with the magnitude of the transformation.^89^ Similarly, tuning of face cells evolves from being view specific to being invariant for mirror symmetry and, finally, to being fully view invariant along the posterior-anterior progression of face patches in monkey IT, with a concomitant increase of response latency.^90^ Finally, it has been shown that more challenging object discriminations require longer processing time by IT neurons and can only be modeled by either very deep feedforward CNNs or shallower, but recurrent, networks.^91^^,^^92^

Yet another factor that could contribute to explain the large tolerance of rats to image manipulations as severe as partial occlusion is their low visual acuity. In fact, experiencing a blurred visual world may yield visual representations that are intrinsically more robust to various forms of noise, as recently shown for deep CNNs by Jang and Tong.^76^ Our tests with their VGG-16 trained in the strong-blur regime confirmed this intuition, showing that, for this network, the best match with rat perception of the partially occluded views took place in earlier convolutional layers (Figure 9B, right), as compared to the standard VGG-16 trained with high-resolution images from ImageNet (Figure 4D).

As for generalization to outlines, the struggle of VGG-16 to deal with such image manipulation is fully consistent with previous studies showing how convolutional networks are much less proficient than humans in recognizing objects that are deprived of texture, such us silhouettes and outlines (with the latter being particularly challenging for CNNs).^93^^,^^94^^,^^95^ This indicates that CNNs process images by exploiting sets of features that are substantially different from those used by humans, i.e., without extracting a representation of the global shape of visual objects^39^^,^^94^ but heavily relying instead on texture information.^95^ By contrast, rat proficiency in dealing with outlines indicates an intriguing similarity in the way rats and humans process visual shapes.

Taken together, these considerations suggest that the merit of the layer-by-layer comparison between CNNs’ representations and rat object vision lies in the possibility to bridge rat and human visual perception. Which depth of a CNN yields the best match with rat discrimination accuracy does not matter much per se. It is not surprising that CNNs largely surpass rats in terms of performance magnitude in object-recognition tasks—they do so with human performance also.^96^ The more intriguing question is whether the way visual perception departs from the processing carried out by CNNs is similar in rats and humans. Our findings indicate that this is indeed the case, and to a surprising extent. Both humans and rats display similarly high robustness to heavy image occlusion and reduction of objects to outlines, a robustness that CNNs struggle to achieve. Eberhardt and colleagues^27^ have shown that, when humans are tested in a speeded image-classification task, their accuracy is surpassed by the networks of the VGG family already in early to mid-level layers, while the correlation between human and CNN confidence in classifying the images peaks in mid- to late-level layers.

The consistency of these findings with those of our experiments (Figures 3E, 3F, 4B, and 4C) suggests that the core mechanisms underlying shape processing in humans and rats are similar and are similarly different from those of CNNs. In the spirit of what is suggested for humans,^87^^,^^97^ rats, under the pressure of the discrimination task and of the water-restriction regime, likely process incoming visual objects via a fast feedforward sweep through the visual hierarchy. This allows them to achieve performances that are high enough (even in the face of translation, rotation, and scaling) to fulfill the goal of obtaining the liquid reward as fast as possible (note that achieving very high accuracy on individual stimulus presentations at the cost of a slower response time and, therefore, of a lower number of stimulus presentations, may not be optimal in terms of the overall amount of reward achieved over the course of the experiment). However, the animals, as postulated for humans and suggested by monkey studies, when facing much more challenging image manipulations (such as occlusion and outlines), may flexibly increase the depth of visual processing by engaging recurrent processes^86^^,^^87^^,^^91^^,^^92^^,^^97^^,^^98^—a strategy that, in standard CNNs, would require exploiting the full depth of the purely feedforward architecture.

The existence of systematic differences in the processing of shape information between CNNs and biological brains was confirmed by the comparison we carried out at the level of perceptual strategies—the third main finding of our study. As already shown by Alemi-Neissi and colleagues,^5^ rat perceptual strategy, as revealed by the animals’ responses to partially occluded images, was quite consistent with that of an ideal observer, which, by design, was optimally tolerant to object transformations (Figures 5 and 6A). By contrast, rat strategy correlated poorly with the saliency maps extracted across VGG layers, which, not surprisingly, were also poorly consistent with those of the ideal observer (Figure 6A). More importantly, we found that the diagnostic features used by rats to recognize visual objects across view changes were considerably more invariant than those afforded by the representations of the same objects along VGG layers, even the deepest ones (Figures 6C and 6D), a conclusion that was confirmed by comparing the saliency maps yielded by radically different appearances of visual objects, such as full bodies vs. outlines (Figure 7D).

These findings, taken together with the results discussed in the previous paragraphs, suggest the existence of a trade-off between the discrimination accuracy a given representation can afford and the invariance of the set of visual features encoded by the representation upon which the discrimination is based. VGG-16 deep layers support much higher classification accuracies of the unoccluded stimuli as compared to rat performance. However, these larger accuracies are attained by relying on object representations that are more view specific than the perceptual templates exploited by the rats. This seems consistent with the ability of deep CNNs to achieve virtually perfect training accuracy in classification tasks where images are randomly associated to arbitrary category labels^99^—an impressive feat that, however, points to a fundamental difference from biological vision in terms of the ability to learn abstract category information.^39^^,^^87^ Under this scenario, it is unclear which of the two systems actually encode objects in the most “advanced” way. Despite the larger accuracy it affords, the stronger view dependency of VGG-16 representations could be at the root of their lower ability to generalize to severe, out-of-distribution, image-level manipulations (such as occlusion and reduction to outlines).

These considerations are supported by the tests we carried out with an untrained VGG-16 (Figures 8D–8F) and with the network trained with strongly blurred images (Figures 9C–9E). In both cases, the object-centered view invariance of the discrimination strategies increased as compared to that of the standard VGG-16 pre-trained on ImageNet (compare to Figures 5 and 6). This suggests that the lack of training, as well as the robustification granted by the training regime with low spatial frequencies, yield representations that are less specialized to process specific image sets and, as such, better allow “discovering” the same features across different object views. On the other hand, the saliency maps obtained for these networks departed substantially from those of the rats, becoming anti-correlated with rat perceptual strategies and with those of the ideal observer model. This means that the rat visual system is able to extract those shape features that are more discriminatory of object identity across view changes in a way that is unmatched by any of the networks we tested.

Interestingly, these conclusions resonate with those of recent studies showing that the object-recognition strategies deployed by deep nets are poorly aligned with those used by human observers and with the saliency maps describing the tuning of IT neurons.^36^^,^^37^^,^^38^ Moreover, such discrepancy has worsened as the discrimination accuracy on ImageNet of more recent (and larger) architectures has increased. This has led some authors to design a training routine to explicitly align the visual strategies of deep nets with those employed by humans, with the goal of obtaining more biologically relevant models of visual perception and cortical processing.^37^^,^^38^ The importance of matching perceptual strategies between biological visual systems and neural networks is also highlighted by another study,^100^ where a generative model of visual faces was used to parametrically control various properties of face features. The authors tested different neural network models in terms of their ability to predict human face similarity judgments, showing that the best models shared with humans a consistent set of diagnostic face landmarks. Yet other studies have tackled the same issue by complementing the training diet of CNNs with the addition of images where texture had been altered in such a way as to be uninformative about category labels^95^ or where blurring had been added to mimic the low resolution of peripheral retina.^76^ As a result of this augmentation of the visual diet, CNNs acquired a stronger sensitivity to the shape of visual objects as opposed to their texture, thus matching more closely human perception and also improving in terms of robustness to various forms of noise and distortions.

In the light of these considerations, the results of our study not only indicate a surprising degree of consistency between species so evolutionary and ecologically distant as rodents and primates but also reinforce the need to further increase the robustness, generalizability, and biological consistency of CNNs.^35^^,^^39^ For instance, in the spirit of Jang and Tong^76^^,^^95^ and Geirhos et al.,^76^^,^^95^ our study suggests that images with a variable amount of occlusion (e.g., via bubbles masks) should be included in the training diet of CNNs to make them more robust to such manipulation. Also, in the spirit of Linsley et al.^37^^,^^38^ and Fel et al.,^37^^,^^38^ a high degree of consistency between the visual features used to recognize augmented versions of the same objects could be enforced in the training routine of CNNs with the goal of learning more invariant shape representations of whole objects. Finally, in the spirit of Rajaei et al.,^86^^,^^87^^,^^98^^,^^101^ Kreiman et al.,^86^^,^^87^^,^^98^^,^^101^ Spoerer et al.,^86^^,^^87^^,^^98^^,^^101^ and Kietzmann et al.,^86^^,^^87^^,^^98^^,^^101^ our study suggests that some amount of recurrent connectivity should be included in CNN architectures to allow adjustment of the depth of visual processing by flexibly selecting the number of repeated sweeps through the processing hierarchy, depending on the needs of the task at hand.

In summary, our work not only reasserts the sophistication of rat vision and, therefore, the importance of rodent models in dissecting the neuronal circuits underlying visual perception, but it also points to potentially interesting adjustments to the architecture and visual diet of CNNs to bring their representations, robustness, and generalization power closer to their biological counterparts.

## Methods

### Computation of the contrast-sensitivity function

The CSF (see Figure 2A) provides a concise description of the acuity of an organism’s visual system by assessing the contrast visibility threshold of static sine-wave gratings as a function of the stimulus’ spatial frequency. Operationally, the CSF can be estimated by administering a visual detection task, whereby an observer is required to report whether sine-wave gratings of variable spatial frequencies and contrast are detectable over a gray background. By letting the contrast *c* vary from full visibility (i.e., the condition in which the difference between maximal and minimal luminance is the greatest) to no visibility (i.e., uniform gray display), one can reconstruct the psychometric response functions p(detection,c;ν), where *p* is the probability of detecting the grating as a function of its contrast *c*, given a spatial frequency ν (see Figure 2C). The contrast sensitivity threshold ξ(ν) can be defined in different ways.^30^^,^^102^^,^^103^ The simplest approach is to set it to the contrast value yielding a criterion detection performance and is often chosen as the point of subjective equality—i.e., *ξ* such that p(detection,ξ;ν)=50% (dashed red line in Figure 2C). The contrast sensitivity σ(ν) (at a specific spatial frequency) is then computed as the reciprocal of the measured sensitivity threshold, i.e., σ(ν)=1/ξ(ν). The CSF is thus estimated by measuring σ(ν) for different values of the spatial frequency ν of the gratings. This approach was used to compute the CSFs of the simulated observer shown in Figure 2A (purple curves), as explained in results.

### Image-augmentation pipeline

The image-augmentation pipeline simulates the additional image variability produced by the head movements of the rats that, in our experiments, were not head fixed but only slightly body restrained. In fact, the animals had to insert their head through a viewing whole in order to face the stimulus display and get access to the array of touch sensors that allowed them to trigger stimulus presentation and report stimulus identity.^4^^,^^5^^,^^12^ This ensured precise control over the viewing distance *d* (which, in all our experiments, was set to d=30 cm), but allowed for variability in head rotation around the three Euler angles (yaw, pitch, and roll) at the time when the rat sampled the visual stimulus. The images were shown on a monitor of size 48cm×27cm (width × height), resulting in a size of 35° visual angle for the default views of the visual objects.

To estimate the impact of head rotation on the appearance of the visual objects used in our experiments, we leveraged previous work from our lab,^32^ in which we characterized the variability of head orientation about the three Euler angles (for rats tested under equivalent experimental conditions as those used in Zoccolan et al.,^4^^,^^5^^,^^12^ Alemi-Neissi et al.,^4^^,^^5^^,^^12^ and Djurdjevic et al.^4^^,^^5^^,^^12^) by means of head tracking. Specifically, our previous study estimated the following ranges of variations of head angles: 60°, 35°, and 20° for pitch, roll, and yaw, respectively. This resulted in vertical translations (pitch), horizontal translations (yaw), and in-plane rotations (roll), the extent of which we quantified based on the geometry of the experimental setup.

The magnitude of the in-plane rotations directly corresponds to the magnitude of the roll rotations $\theta_{r}$. Therefore, in our augmentation pipeline, we assigned to any visual object fed to VGG-16 a random in-plane rotation $\alpha =\theta_{r}$, with $\theta_{r}$ sampled uniformly in $∼\left[-17.5^{\circ },+17.5^{\circ }\right]$. To quantify the horizontal and vertical displacements $\Delta_{H|V}^{\text{cm}}$ of object images due to variations in pitch $\theta_{p}$ and yaw $\theta_{y}$, we computed

(Equation 1) $$\Delta_{H|V}^{\text{cm}}=2d\;\tan\left(\frac{\theta_{p|y}}{2}\right)\text{.}$$

The resulting $\Delta_{H|V}^{\text{cm}}$ is expressed in cm. However, for our image-augmentation pipeline, we used the PyTorch transforms.RandomAffine routine, which expects the *x*-*y* translations to be specified as a fraction of the original image size. We thus made a conversion from cm to pixel-count by knowing $S_{H|V}^{\text{cm}}$, the size of the images presented to the rats in cm, and $R_{H|V}^{\text{pix}}$, the screen resolution in pixels, and by computing the cm-to-pixel conversion factor as $\rho_{\text{cm}}^{\text{pix}}=R_{H|V}^{\text{pix}}/S_{H|V}^{\text{cm}}$. One can then express both the image size and the displacement in pixels as, respectively, $I_{H|V}^{\text{pix}}=\rho_{\text{cm}}^{\text{pix}}S_{H|V}^{\text{cm}}$ and $\Delta_{H|V}^{\text{pix}}=\rho_{\text{cm}}^{\text{pix}}\Delta_{H|V}^{\text{cm}}$. The *x*-*y* translations were then uniformly sampled as $x∼\left[-\frac{\Delta_{H}^{\text{pix}}}{2I_{H}^{\text{pix}}},+\frac{\Delta_{H}^{\text{pix}}}{2I_{H}^{\text{pix}}}\right]$ and $y∼\left[-\frac{\Delta_{V}^{\text{pix}}}{2I_{V}^{\text{pix}}},+\frac{\Delta_{V}^{\text{pix}}}{2I_{V}^{\text{pix}}}\right]$. The full augmentation pipeline encompassed all the steps described above (see examples in Figure 2E) and included also the addition of Gaussian blur and random Gaussian noise, as explained in the results section (see Figure 2).

### Training and testing procedure of the linear SVMs on the object representations yielded by VGG-16 layers

In all our experiments, we used a VGG-16 network (from PyTorch) pre-trained on the ImageNet dataset. For each convolutional and fully connected layer of the network, we built a linear SVM classifier (using the sklearn.svm Python implementation) that was trained to predict the identity (i.e., the label) of the visual objects used in the rat experiments, using the activations of a subpopulation of units measured before the non-linear rectified linear unit (ReLU) gate. We measured these activations for all the units in a layer, following the presentation of the (augmented) stimulus conditions of a given rat experiment, and then we sampled a random subpopulation with a given size. We explored the following range of population sizes: $\left\{10^{3},5\times 10^{3},10^{4},5\times 10^{4},{\text{and}\;10}^{5}\right\}$. Scaling beyond this value was limited by memory constraints. However, not all VGG-16 layers have a total unit count greater than the biggest scale considered (for instance, the output layer only has $10^{3}$ units). In the case when the total number of units in a layer was smaller than the considered scale, we simply took the whole population of units. This only occurred for the linear, fully connected layers, as the last convolutional layer in VGG-16 has more than $10^{5}$ units.

Before being fed to the linear classifier, the sampled activations were independently normalized to have zero mean and unit variance across the set of stimulus conditions used for training (this was done with the scaler implementation from sklearn.preprocessing.StandardScaler). Thanks to the augmentation pipeline explained in the previous section, we could scale the number of both training and testing conditions beyond the original sets described in the rat studies simply by resampling from each set and exploiting the additional variability introduced by the random augmentations. It should be noted that this procedure was adopted to not only to achieve a more robust training/testing of the classifiers but also because it more closely reflected the actual amount of image-level variability experienced by the rats.

In all experiments, we trained the linear classifiers on $5\times 10^{3}$ images sampled from the appropriate stimulus set, while the number of testing conditions varied based on the rat study under examination. In the experiments where the stimulus set of Zoccolan and colleagues^4^ was used, we tested the SVMs on $5\times 10^{3}$ images. As explained in the results, the training and testing conditions were, respectively, the on-cross and off-cross stimuli shown in Figure 3A (red frames). In the experiments where the images from the study of Alemi-Neissi and colleagues^5^ were used, we trained all the SVMs on a mixture of unoccluded and partially occluded images, with the same probability of occurrence (50%) for each image type. While in Alemi-Neissi and colleagues^5^ only a few selected object views were presented in their occluded version (marked by the red stars in Figure 4B), the SVMs, during training, were exposed to partially occluded views of all the object transformations included in the stimulus set (i.e., all variations in size, azimuth rotation, horizontal shift, and in-plane rotation shown in the axes of Figure 4B). As explained in the results, this was necessary, because otherwise the generalization of the SVMs to the occluded views was very poor and did not allow recovery of the saliency maps for all combinations of stimulus conditions and layers (see next section). For the accuracy analysis shown in Figure 4B, we tested separately $2.5\times 10^{3}$ unoccluded and $2.5\times 10^{3}$ randomly occluded images. For inferring the saliency maps underlying the SVMs’ visual strategies, the number of test conditions is reported in the next section. Finally, in the experiments where the stimulus set of Djurdjevic and colleages^12^ was used, we tested the SVMs with $2.5\times 10^{3}$ images separately for both the regular and outline object conditions (see Figure 7).

In the experiments with the stimulus set of Alemi-Neissi and colleagues,^5^ we additionally trained a pixel-based classifier in the object discrimination task originally administered to the rats (cyan curves/stars in Figure 4B). Also in this case, we used a linear SVM classifier (using sklearn.svm) and trained it to predict the object labels using the full set of pixels (224×224) as input features. For consistency with the experiments done with VGG-16, we trained the pixel-based classifier with $5\times 10^{3}$ images, using an even mixture of unoccluded and occluded images sampled from the entire stimulus set, and we tested it with $2.5\times 10^{3}$ images separately for each image type.

All classification experiments were repeated five times (referred to in the results as classification runs). In each run, we performed a full resampling of the subpopulations of units in each VGG-16 layer, whose activations were fed to the linear SVMs. In addition, in each run, all training and test images underwent different random augmentations.

### Visual strategy identification

We used two different approaches to infer the visual strategies used by the SVM classifiers, trained with VGG-16 layer-based activations. The first was based on the classification of partially occluded views of the visual objects and the second on the classification of random structural variations of a target object. Both approaches closely matched the image classification methods used, respectively, by Alemi-Neissi and colleagues^5^ and Djurdjevic and colleagues^12^ to uncover rat visual perceptual strategies.

In the first approach, known as the bubbles method,^57^ partially occluded images of each object view are obtained by imposing a random mask $m_{ij}\in \left[0,1\right]$ on top of the image, with i,j being spatial indices indicating the pixel coordinate in the image. Each original pixel $p_{ij}$ is alpha-masked via $p_{ij}^{*}={\text{bkg}}_{ij}\left(1-m_{ij}\right)+p_{ij}m_{ij}$, where we take ${\text{bkg}}_{ij}$ to be a uniform black background. The occluding mask $m_{ij}$ is constructed by randomly sprinkling $N_{b}$ Gaussians of fixed variance *σ* (the bubbles) in a region centered on the object and spanning 70% of the total image size. In our experiments, we fixed $N_{b}=40$ and set *σ* such that the resulting bubbles had a size equivalent to $2^{\circ }$ of visual angle. These values were selected based on the experimental design of Alemi-Neissi et al.,^5^ who used bubbles of $2^{\circ }$ of visual angle, while $N_{b}$ was rat specific and was chosen so as to bring each rat performance to be 10% lower than with the unmasked objects. Since $N_{b}$ ranged between 10 and 90, depending on the proficiency of the rat, in our tests with VGG-16 we set $N_{b}=40$, as an intermediate rat value. In our experiments, image masking occurred before the random image augmentation described previously.

To derive the visual strategy of the observer for a particular object view, one has to compute the classification labels $l_{\mu }\in \left\{0,1\right\}$ (where 0 represents an error and 1 represents a correct choice) for a set of masked images ${\left\{m_{ij}^{\mu }\right\}}_{\mu }^{N_{m}}$, where *μ* is the index of the specific mask. Following Alemi-Neissi and colleagues,^5^ we took $N_{m}=3000$. The saliency map $S_{ij}$ obtained for the object view can then be reconstructed as

(Equation 2) $$S_{ij}=\frac{\sum_{\mu }m_{ij}^{\mu }l_{\mu }}{\sum_{\mu }\left|m_{ij}^{\mu }\right|}\text{.}$$

To assess whether the saliency value $S_{ij}$ computed for a given pixel was significantly correlated with the choices of the observer, we performed the following permutation test. We sampled 1,000 random permutations of the classification vector, obtaining $l_{\mu }^{\left(\nu \right)}$, with ν∈{0,1,…,1,000}. We next applied Equation 2 using $l_{\mu }^{\left(\nu \right)}$ to estimate the null distribution $S_{ij}^{\left(\nu \right)}$. We then used a one-tailed statistical test to find which pixel values $S_{ij}$ were statistically higher than expected by chance (i.e., with $p<p_{\text{th}}$) according to these null distributions $S_{ij}^{\left(\nu \right)}$, in such a way to identify significantly salient regions. Similarly, significantly anti-salient regions were identified as the sets of pixels for which $S_{ij}$ was statistically lower (i.e., with $p<p_{\text{th}}$) than expected by chance. For a visual comparison among the saliency maps obtained for the rats and the SVMs trained on VGG-16 layer-based representations, we set $p_{\text{th}}=0.05$, i.e., the same value used by Alemi-Neissi and colleagues^5^ (this threshold yielded the salient and anti-salient regions highlighted by the red and cyan patches in Figure 5). Instead, for the overlap analysis shown in Figure 6, $p_{\text{th}}$ in the saliency maps obtained for the SVMs was independently adjusted for every tested object view and each layer of the network in such a way that the overall area of the salient region matched the one of the average saliency map obtained for the rat. This ensured that any difference in the amount of overlap between the maps obtained for different object views was not merely due to the different size of the salient regions being measured for the rats and the SVMs.

The second approach, introduced by Djurdjevic and colleagues,^12^ follows a different experimental paradigm: the subject is trained to recognize a given target object against a set of “distractor” objects. Once the classification is learned, the subject enters a testing phase where is exposed to random shape variations $T_{ij}^{\mu }$ of the target object, and its spontaneous choices $r_{\mu }\in \left\{0,1\right\}$ are collected (here 0 indicates that the random variant *μ* is classified as a distractor, while 1 indicates that is classified as being the target object). The saliency map associated to the target object can then be estimated (as illustrated in Figure 7A) as

(Equation 3) $$S_{ij}=\underset{\mu :r_{\mu }=1}{E}\left[T_{ij}^{\mu }\right]-\underset{\mu :r_{\mu }=0}{E}\left[T_{ij}^{\mu }\right]\text{.}$$

Also in this case, the statistical significance of the pixels in the saliency map was estimated via a permutation test. We produced 100 random permutations of the observer’s classification vector $r_{\mu }^{\left(\nu \right)}$, with ν∈{0,1,…,100}, from which we estimated the corresponding null distributions of saliency values $S_{ij}^{\left(\nu \right)}$ , by applying Equation 3 using $r_{\mu }^{\left(\nu \right)}$. For each pixel, we then fitted a one-dimensional Gaussian to obtain a smooth version of the null-distribution. Finally, using the estimated Gaussian distribution, we performed a one-tailed test by comparing where the pixel values of the original saliency map (i.e., those obtained via the actual subject response vector $r_{\mu }$) sit with respect to the null distributions: a pixel was deemed significantly salient if it fell on the positive tail (p<0.01) and significantly anti-salient if it fell on the negative one (p<0.01).

### Parameter-matched multi-layer perceptron

In our control experiment on architectural prior benefits on classification accuracy, we developed a multi-layer perceptron (MLP) that was simultaneously depth- and parameter-matched with our reference network, i.e., VGG-16. The latter employs a stack of d=16 layers and leverages a total of P∼138 million internal parameters, which we took as our two targets to match. We selected a simple architecture composed of a cascade of linear (i.e., fully connected) layers, each followed by a non-linear activation gate, which we set to be the commonly employed ReLU. We could thus functionally represent the MLP network simply as

(Equation 4) $$\text{MLP}\equiv M^{\left(d\right)}\circ \text{ReLU}\circ M^{\left(d-1\right)}\circ \text{ReLU}\circ \cdots \circ M^{\left(1\right)},$$

where we set the depth d=16, and each $M^{\left(l\right)}\in R^{N_{l}\times N_{l-1}}$ represents the linear matrix multiplication operation going from layer l−1 of dimensionality $N_{l-1}$ to layer l of dimensionality $N_{l}$. Having fixed *d*, the task was to select the layer dimensionalities ${\left\{N_{i}\right\}}_{i}$ such that the total parameter count equaled *P*, with the additional constraint that $N_{0}$ is fixed by the size of the input, which in our case is the image resolution at 224×224 (we purposely excluded the redundant channel dimension given our black-and-white-only image distribution to avoid unnecessarily inflating the parameter count at the input layer). We additionally fixed the output dimensionality $N_{d}=1,000$ to equal the one of VGG-16 and set the remaining values $N_{i}$ according to the full specification reported in Table 1. We implemented the MLP network in Python using the torch library. All network layers were randomly initialized following the default random initialization setup provided by the torch.nn.Linear Python library.

**Table 1:** Complete architecture specification of the depth- and parameter-matched multi-layer perceptron

Layer	Input dim. ($N_{l-1}$)	Output dim. ($N_{l}$)	Parameter count ($10^{6}$)
$M^{\left(1\right)}$	224×224	2,150	108
$M^{\left(2\right)}$	2,150	2,048	4
$M^{\left(3\right)}\to M^{\left(5\right)}$	2,048	2,048	4 (×3)
$M^{\left(6\right)}$	2,048	1,024	2
$M^{\left(7\right)}\to M^{\left(15\right)}$	1,024	1,024	1 (×9)
$M^{\left(16\right)}$	1,024	1,000	1
Total parameter count			136

## Resource availability

### Lead contact

Further information and requests for resources should be directed to and will be fulfilled by the lead contact, Davide Zoccolan (zoccolan@sissa.it).

### Materials availability

This study did not generate new unique reagents.

### Data and code availability

All of the (Python) analysis code and the image datasets necessary to run such analyses are publicly available in a Zenodo repository. Original data have been deposited to Zenodo Data: https://doi.org/10.5281/zenodo.13987011.^104^

## Acknowledgments

This work was funded by the European Union – NextGenerationEU – PNRR M4C2-I.1.1, in the framework of the PRIN Project no. 2022WX3FM5, CUP: G53D23003220006 (D.Z.). The views and opinions expressed are solely those of the authors and do not necessarily reflect those of the European Union, nor can the European Union be held responsible for them. The study was also supported by the Italian Ministry of University and Research under the call PRO3, project NEMESI (D.Z.); the 10.13039/100000893Simons Foundation Collaboration on the Global Brain grants 542989SPI and NC-GB-CULM-00002734 (A.A.); and 10.13039/100000002NIH grant R01 NS104926 (A.A.). We thank Alessio Ansuini for easing access to data of Djurdjevic et al.^12^ and Laura Porta for her contribution to initial pilot tests with VGG-16. We also thank Gabriel Kreiman, Fabio Anselmi, Sebastian Goldt, and Alessandro Sanzeni for valuable discussions and insightful feedback on our manuscript.

## Author contributions

Conceptualization, P.M. and D.Z.; methodology P.M., A.A., and D.Z.; software, P.M.; formal analysis, P.M. and D.Z.; investigation, P.M. and A.A.; resources, A.A. and D.Z.; data curation, P.M. and A.A.; writing – original draft, P.M. and D.Z.; writing – review & editing, P.M., A.A., and D.Z.; visualization, P.M.; supervision, D.Z.; funding acquisition, D.Z.

## Declaration of interests

The authors declare no competing interests.

## Supplemental information

Document S1. Figure S1

Document S2. Article plus supplemental information
