# Supplementary material for: Unraveling the complexity of rat object vision requires a full convolutional network and beyond
Source: Patterns (N Y). 2025 Jan 17;6(2):101149. doi: 10.1016/j.patter.2024.101149 (PMC11873012; doi:10.1016/j.patter.2024.101149)
Supplement: Document S1. Figure S1 [file mmc1.pdf]

Patterns, Volume *6*

Supplemental information

Unraveling the complexity of rat object vision requires a full convolutional network and beyond

Paolo Muratore, Alireza Alemi, and Davide Zoccolan

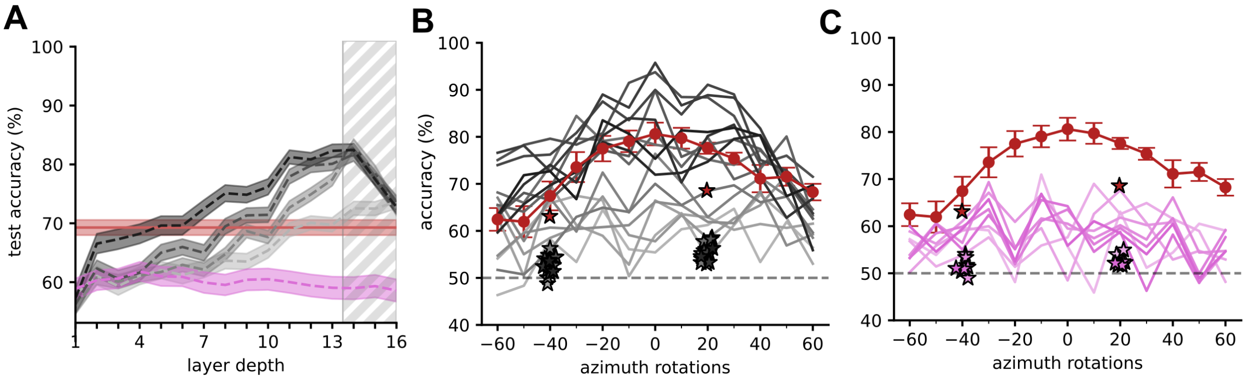

Figure S1: **Comparing rat discrimination accuracy with that of an unstrained VGG-16 and an unstrained multilayer perceptron.** (**A**) Same analysis as in Figure 3D, but for a randomly-initialized VGG-16 (shades of gray) and a randomly initialized multilayer perceptron (MLP; pink). Same colors and symbols as in Figure 3D. (**B-C**) Same analyses as in Figure 4B (top-right plot), but for a randomly-initialized VGG-16 (**B**) and a randomly initialized MLP (**C**). Same colors and symbols as in Figure 4B.
